# Biodiversity pattern of fish assemblages in Poyang Lake Basin: Threat and conservation

**DOI:** 10.1002/ece3.5661

**Published:** 2019-09-26

**Authors:** Xiongjun Liu, Jiajun Qin, Yang Xu, Min Zhou, Xiaoping Wu, Shan Ouyang

**Affiliations:** ^1^ Poyang Lake Key Laboratory of Environment and Resource Utilization School of Resource, Environment and Chemical Engineering Ministry of Education Nanchang University Nanchang China; ^2^ School of Resource, Environment and Chemical Engineering Nanchang University Nanchang China; ^3^ School of Life Sciences Nanchang University Nanchang China; ^4^ School of Foreign Languages Nanchang University Nanchang China

**Keywords:** beta diversity, biodiversity, conservation, fish, Poyang Lake Basin

## Abstract

Poyang Lake Basin is of great importance to maintain regional ecological balance. However, fish biodiversity in this basin has rapidly declined as the result of anthropogenic habitat alteration, such as dam construction, sand mining, and water pollution. Here, we aimed to analyze the temporal and spatial changes in biodiversity patterns of fish in Poyang Lake Basin over the last 37 years. The number of fish species underwent a significant decrease in the current period. In particular, 36.7% of the migration of fish was extirpated. Twenty‐seven fish species have been formally assessed using the Chinese Red List were currently listed as Critically Endangered (9), Endangered (3), Vulnerable (10), and Near Threatened (5). Alpha and gamma diversity revealed that fish diversity had also decreased, and beta diversity showed significant composition dissimilarity in two periods. PCoA showed that the historical fish composition dissimilarity was significantly different from that of the current period. We found a significant effect of the geographical distance on the spatial turnover component for the historical and current periods. In addition, the nestedness component was the main contributor to beta diversity, which indicated one large protected area should be established in Poyang Lake and the Ganjiang River Basin with higher species richness. These results indicated that fish biodiversity declined in the current period likely caused by anthropogenic habitat alteration and other threatened factors. Therefore, we suggest that the habitat reconstruction and biodiversity conservation for fish have become imperative in this basin, and a complete management plan should be carried out.

## Graphical abstract

Poyang Lake Basin is of great importance to maintain regional ecological balance. However, fish diversity in this basin has rapidly declined as the result of human activities. Here, we aimed to analyze how temporal and spatial changes impact biodiversity patterns of the fish assemblages in Poyang Lake Basin over the last 37 years. The diversity of fish species underwent a significant decrease. In particular, 36.7% of the migration of fish was extirpated. Twenty‐seven fish species have been formally assessed using the Chinese Red List were currently listed as Critically Endangered (9), Endangered (3), Vulnerable (10), and Near Threatened (5). Alpha and gamma diversity revealed that fish diversity had also decreased, and beta diversity showed a significant trend toward taxonomic homogenization. PCoA showed that the historical fish composition similarity was significantly different from that of the current period. These results indicated that human activities have seriously affected the fish habitat and led to the decline in fish diversity.

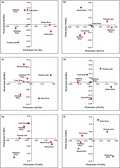

## 1 INTRODUCTION

Species compositions become gradually more similar (i.e., biological homogenization) as a result of anthropogenic habitat alteration, such as hydrologic alteration, habitat fragmentation, overfishing, eutrophication, and species invasion, which result in an increasing risk of extinction in certain current species (Cardinale et al., 2012; Monnet et al., 2014; Olden, Comte, & Giam, 2016; Taylor, 2010). Biological homogenization refers to disparate regions becoming more similar in their species compositions through time (Olden & Rooney, 2006), and beta diversity is defined as the variation in species composition between sites (Whittaker, 1960). In other words, biological homogenization is the process by which beta diversity decreases over time. For more than a decade, considerable evidence has been accumulated indicating a general trend toward biotic homogenization in various taxa in terrestrial, freshwater, and marine ecosystems across the earth (e.g., Qian & Ricklefs, 2006; Toussaint, Beauchard, Oberdorff, Brosse, & Villeger, 2014; Winter et al., 2009).

Freshwater fish can be indicators of the aquatic ecosystem quality (Arthington, Dulvy, Gladstone, & Winfield, 2016; Nogueira et al., 2010; Yan, Xiang, Chu, Zhan, & Fu, 2011) and are also a rich source of nutrition, constituting a major staple food item for most people (Cressey, 2009; De Silva, 2012; Naylor et al., 2000). However, due to the effect of anthropogenic habitat alteration, the composition of fish species has become progressively more similar, and endemic species have been continuously globally threatened (Arthington et al., 2016; Fu, Wu, Chen, Wu, & Lei, 2003). Therefore, freshwater fish are considered to be among the most vulnerable groups of organisms, as many of these species are declining precipitously worldwide (Arthington et al., 2016; Liu, Hu, Ao, Wu, & Ouyang, 2017).

Poyang Lake, the largest lake in China, is important both nationally and internationally because of its geographic position, and it should have priority for conservation efforts because it is also a listed site in the Global Ecoregion 2000 by the World Wildlife Fund (WWF; Fu et al., 2003; Huang, Wu, & Li, 2013). Therefore, Poyang Lake plays an important role in maintaining and supplementing the aquatic biodiversity of the Yangtze River (Jin, Nie, Li, Chen, & Zhou, 2012). However, there have been serious negative impacts on fish biodiversity because of human activities. Many fish species are assessed as threatened or near threatened based on the Chinese Red List (Jiang et al., 2016). In addition, some species of fish are likely to be extirpated from Poyang Lake Basin, such as *Acipenser sinensis* Gray, 1835, *Psephurus gladius* (Martens, 1862), *Ochetobius elongatus* (Kner, 1867), *Tenualosa reevesii* (Richardson, 1846), and *Luciobrama macrocephalus* (Lacepède, 1803).

Knowledge on the biodiversity patterns of fish is important for proposing conservation and management strategies. However, few studies have compared changes in the biodiversity patterns of fish in the historical and current periods, Poyang Lake Basin. Beta diversity is an important tool for conservation planning (Bergamin et al., 2017; Mcknight et al., 2007; Wiersma & Urban, 2005). The objective of this study was to analyze the temporal and spatial changes in the biodiversity patterns of fish and to explore the effects of geographical factors on the biodiversity patterns. We hope our study provides an important basis for the conservation and management of fish biodiversity.

## 2 METHODS

### 2.1 Study area

**Figure 1:**
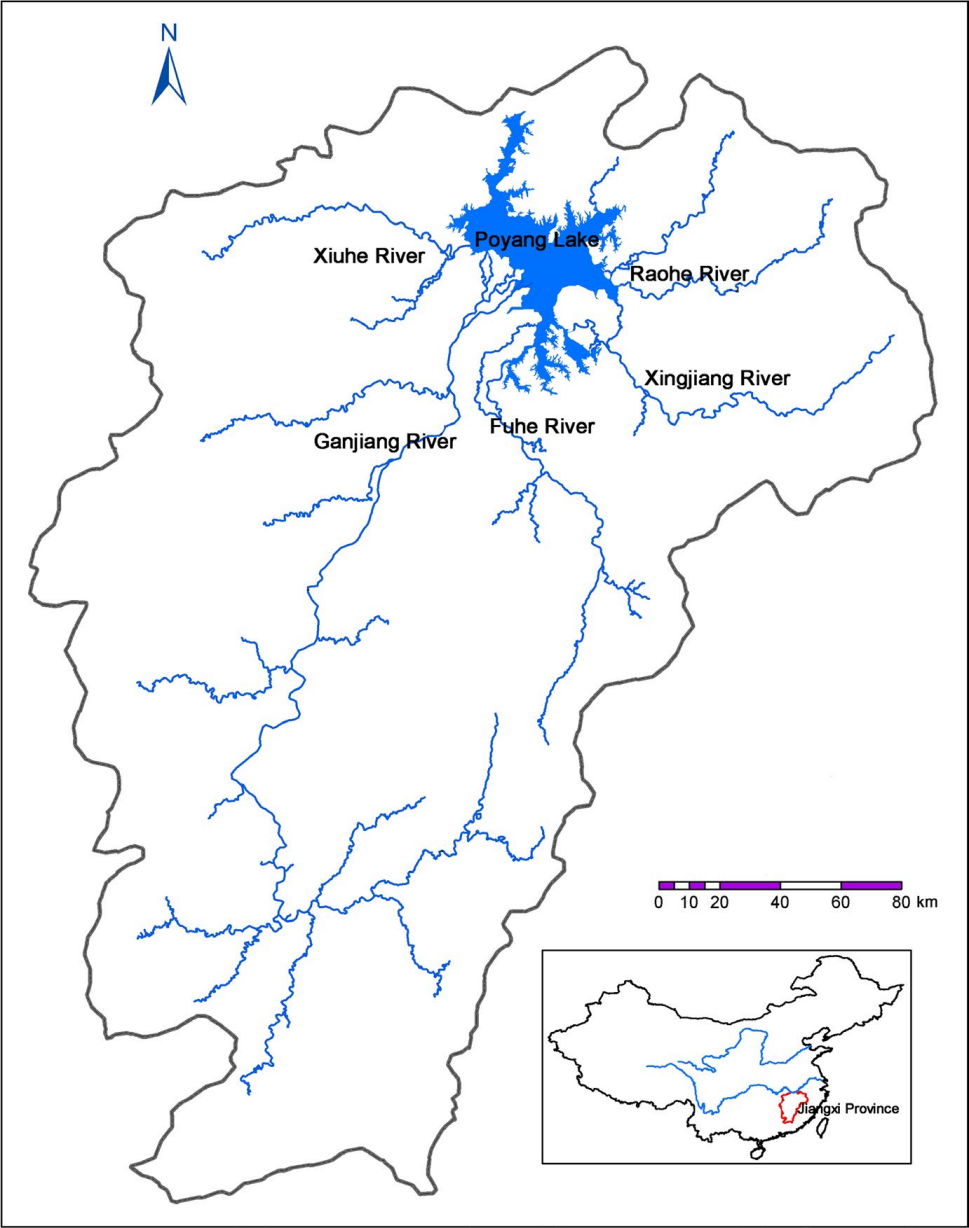
Map showing the location of Poyang Lake Basin

Poyang Lake, located in the north of Jiangxi Province, is surrounded on three sides by mountains, fed by five large rivers (Ganjiang River, Fuhe River, Xiuhe River, Xinjiang River, and Raohe River), and flows into the Yangtze River. Hence, it forms a complex and highly interconnected river–lake–wetland system (Figure 1; Jin et al., 2012). The total area of Poyang Lake Basin is 16.2 × 10^4^ km^2^, accounting for 9% of the Yangtze River Basin and 93.9% of the land area of Jiangxi Province. Poyang Lake Basin has an average annual precipitation of 1,350–2,150 mm, and the precipitation is mainly concentrated in April–June. Its surface runoff is 1,457 × 10^8^ m^3^, accounting for 5.28% of the total runoff in China. An annual average sediment of 2,104.2 × 10^4^ ton flows into Poyang Lake, mainly from the five rivers. The forest coverage in the watershed reaches 60.1%.

### 2.2 Data collection

Since the 1950s, many Chinese researchers have conducted several studies on a wide variety of fish species in Poyang Lake Basin. Lists of the fish species in Poyang Lake Basin have been assembled from published fish surveys (including scientific reports, books, online data, and gray literature) since the 1980s (Table S1). The data provide the most complete account of the freshwater fish distribution in Poyang Lake Basin. FishBase (http://www.fishbase.org/search.php) was used to correct the scientific names of the species from the data. These data were grouped into two periods: (1) the historical period (1980–2000; Table S2) and (2) the current period (2000–2017; Table S3). The published inventories from which the data were taken were based on collections using castnets and electrofishing (most of the surveys before 2000 were conducted using castnets, whereas electrofishing was the main fishing method after 2000). Native and introduced exotic species was distinguished and considered an introduced exotic species as present in the lake only when the species was established.

### 2.3 Data analysis

We assessed the completeness of the fish species using abundance‐based rarefaction as implemented in iNEXT online in each area (Chao, Ma, & Hsieh, 2016). Confidence intervals (95%) were calculated based on 100 bootstrap replications.

Division of ecological types of fish was according to Ye and Zhang (2002) and Institute of Hydrobiology, Chinese Academy of Sciences (1976). Life habits were divided into migration, settlement, and mountain streams; feeding habits were divided into herbivorous, carnivorous, and omnivorous; water layer habitats were divided into upper layer, lower layer, and demersal.

Alpha and gamma diversity represents the richness of a species in a particular region or community and the sum of the species richness in multiple communities, respectively (Legendre & De Cáceres, 2013). We first quantified alpha diversity (species richness in each area) within each period and gamma diversity (total species richness in Poyang Lake Basin).

Baselga (2010) systematically proposes the beta diversity decomposition method based on the Sørensen index (*β*_sor_), which is decomposed into spatial turnover component (*β*_sim_) and nestedness component (*β*_sne_). The decomposition methods are shown as follows:

$$\beta_{\text{sor}}=\frac{b+c}{2a+b+c}$$

$$\beta_{\text{sim}}=\frac{\min\left(b,c\right)}{a+\min\left(b,c\right)}$$

$$\beta_{\text{sne}}=\frac{\left|b-c\right|}{2a+b+c}\times \frac{a}{a+\min\left(b,c\right)}$$

where *a* is the number of common species between two areas, and *b* and *c* are the numbers of species only present in the first and second areas, respectively. Sørensen index ranges from 0 to 1, representing that no species and all species are common among the two areas, respectively.

A principal component analysis (PCoA) was used to analyze the changes in the fish compositions in Poyang Lake Basin (Legendre & Legendre, 2012). PCoA was performed based on R 3.2.0 version (R Development Core Team, 2014) with the “ade4” package (Dray & Dufour, 2007).

Mantel tests (Legendre & Legendre, 2012) with 9999 permutations were used to assess the correlations (Spearman's method) between three pairwise dissimilarity matrices (sørensen index, spatial turnover component, nestedness component) and the pairwise matrices of geographical drivers (geographical distance, drainage area, annual average runoff) for two periods. ArcMap GIS (ESRI) was used to determine the geographic distance in the pairs of areas by measuring the distances along waterways. The drainage areas and annual average runoff of the area pairs were obtained from the Bureau of Hydrology in Jiangxi Province in 2007. R 3.2.0 (R Development Core Team, 2014) was used to perform all analyses based on the packages BETAPART (Baselga & Orme, 2012) and VEGAN (Oksanen et al., 2015).

## 3 RESULTS

### 3.1 Changes in fish species composition

The total number of fish in the historical period (212 species) was greater than the current period (174 species), which indicated the number of fish species experienced a decrease for temporal change in Poyang Lake Basin. Cypriniformes was the most common family, comprising 56.1% (114) and 55.2% (96) of the total number of fish species in two periods, respectively. 37.7% native species were extirpated in the current period, and 16 native species were not recorded in any area from 1980 to 2017 (Table 1). Nine introduced exotic species were established. The species numbers in the Ganjiang River and Poyang Lake were greater than those in the other areas for spatial change (Table 1). The sampling completeness was more than 95% completeness at each area using the Chao I measures estimator. The final slopes of the species accumulation curves for fish in each area were close to asymptotic (Figure S1).

**Table 1:** The number of native species in Poyang Lake Basin during the historical period and the number and percentage of native, extirpated native, and alien fish species during the current period

Basin	Historical		Current		
	Native	Introduced exotic species	Native	Ex‐Na	Introduced exotic species
Poyang Lake	152	0	107	45 (29.6%)	4 (3.6%)
Ganjiang River	181	0	136	45 (24.9%)	8 (5.6%)
Fuhe River	127	0	57	70 (55.1%)	1 (1.7%)
Xinjiang River	138	0	52	86 (62.3%)	1 (1.9%)
Raohe River	85	0	45	40 (47.1%)	3 (6.3%)
Xiuhe River	83	0	77	6 (7.2%)	7 (8.3%)
Total	212	0	165	63	9

Note

The percentages of the extirpated native and introduced exotic species are in brackets.

Abbreviation: Ex‐Na, extirpated native.

### 3.2 Change in the functional taxa

**Figure 2:**
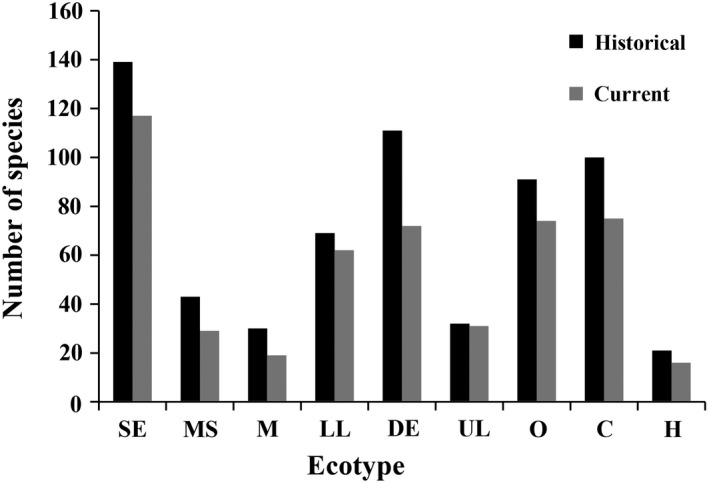
Comparison of the fish ecotypes in the historical and current periods (C, Carnivorous; DE, Demersal fish; H, Herbivorous; LL, Lower‐layer fish; M, Migration fish; MS, Mountain stream fish; O, Omnivorous; SE, Settlement fish; UL, Upper‐layer fish)

25% of carnivores, 18.7% of omnivores, and 23.8% of herbivores were extirpated in the current period, which indicated feeding habits taxa experienced a decrease for temporal change in Poyang Lake Basin (Figure 2). 35.1% of demersal fish, 10.1% of lower‐layer fish, and 3.0% of upper‐layer fish were extirpated in the current period, which indicated habitat characteristics taxa also experienced a decrease (Figure 2). Similarly, 36.7% of migration fish, 32.5% of mountain stream fish, and 15.8% of resident fish were extirpated in the current period, which indicated life habits taxa also experienced a decrease (Figure 2).

### 3.3 Threatened status

The Chinese Red List showed that 65.0% of the species were assessed as Least Concern (LC; Table S4). Twenty‐seven fish species were currently listed as Critically Endangered (9), Endangered (3), Vulnerable (10), and Near Threatened (5) (Table S4).

### 3.4 Changes in the fish diversity

**Figure 3:**
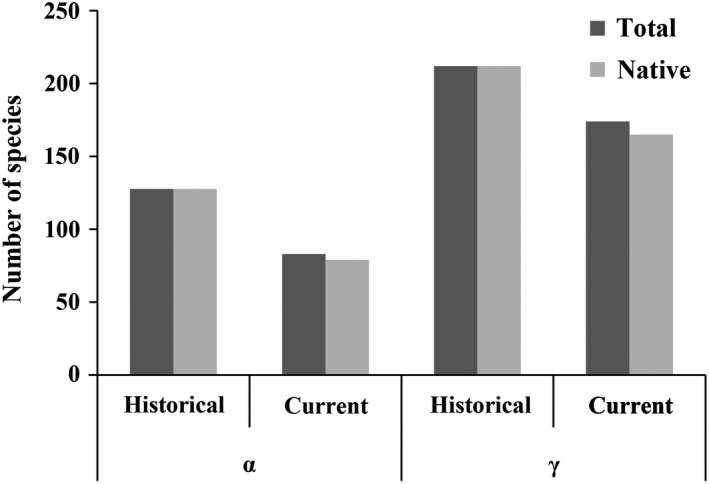
Alpha and gamma diversity of the total fish species and the native species in Poyang Lake Basin during the historical and current periods

Alpha and gamma diversity for all and native species in the current period was lower than the historical period, which indicated fish diversity experienced a decrease (Figure 3). In addition, there was a decrease of alpha and gamma diversity in all river or lake sites experienced in the current period.

A mean value of 0.28 in the historical fish composition dissimilarity among Poyang Lake Basin was lower than the current period (0.40; Table 2). The nestedness component was greater than spatial turnover component for each area in two periods, which indicated that the nestedness component was the main contributor to beta diversity (Table 2).

**Table 2:** Fish compositional dissimilarities during the historical and current periods, as quantified by the Sørensen index (*β*_sor_), and its spatial turnover component (*β*_sim_) and nestedness component (*β*_sne_) in Poyang Lake Basin

Basin	Historical			Current		
	*β*_sor_	*β*_sim_	*β*_sne_	*β*_sor_	*β*_sim_	*β*_sne_
Poyang Lake	0.29 ± 0.07	0.15 ± 0.05	0.14 ± 0.11	0.40 ± 0.07	0.17 ± 0.08	0.23 ± 0.12
Ganjiang River	0.29 ± 0.09	0.07 ± 0.05	0.22 ± 0.13	0.43 ± 0.10	0.10 ± 0.06	0.33 ± 0.16
Fuhe River	0.25 ± 0.05	0.12 ± 0.05	0.13 ± 0.07	0.36 ± 0.07	0.17 ± 0.10	0.19 ± 0.16
Xinjiang River	0.26 ± 0.06	0.13 ± 0.05	0.13 ± 0.09	0.40 ± 0.09	0.20 ± 0.10	0.20 ± 0.17
Raohe River	0.30 ± 0.08	0.10 ± 0.06	0.20 ± 0.13	0.42 ± 0.08	0.18 ± 0.12	0.24 ± 0.19
Xiuhe River	0.31 ± 0.08	0.10 ± 0.05	0.21 ± 0.13	0.37 ± 0.03	0.19 ± 0.07	0.18 ± 0.06
Mean	0.28 ± 0.07	0.11 ± 0.03	0.17 ± 0.12	0.40 ± 0.08	0.17 ± 0.04	0.23 ± 0.13

A total value of all (0.49) and native (0.49) species in the historical fish composition dissimilarity was higher than the current period (Table 3). The spatial turnover component for all and native species was lower than the nestedness component in the historical period, which indicated the nestedness component was the main contributor to beta diversity (Table 3), while the spatial turnover component for all and native species was higher than the nestedness component in the current period.

**Table 3:** Sørensen index (*β*_sor_) and its spatial turnover component (*β*_sim_) and nestedness component (*β*_sne_) for the historical and current periods based on all species, including native species, introduced exotic species, and 27 families

	Historical			Current		
	*β*_sor_	*β*_sim_	*β*_sne_	*β*_sor_	*β*_sim_	*β*_sne_
All species	0.49 ± 0.03	0.24 ± 0.01	0.25 ± 0.02	0.61 ± 0.04	0.32 ± 0.01	0.29 ± 0.03
Native species	0.49 ± 0.04	0.24 ± 0.02	0.25 ± 0.01	0.60 ± 0.06	0.32 ± 0.02	0.28 ± 0.01
Introduced exotic species	0	0	0	0.68 ± 0.07	0.17 ± 0.01	0.51 ± 0.04
Engraulidae	0.57 ± 0.01	0	0.57 ± 0.01	0.67 ± 0.06	0.43 ± 0.05	0.24 ± 0.02
Anguillidae	0.38 ± 0.02	0	0.38 ± 0.02	0.80 ± 0.02	0	0.80 ± 0.02
Cyprinidae	0.45 ± 0.03	0.25 ± 0.01	0.19 ± 0.02	0.57 ± 0.02	0.29 ± 0.01	0.28 ± 0.02
Catostomidae	0.80 ± 0.02	0	0.80 ± 0.02	0.80 ± 0.02	0	0.80 ± 0.02
Cobitidae	0.61 ± 0.04	0.15 ± 0.01	0.46 ± 0.01	0.70 ± 0.02	0.21 ± 0.01	0.49 ± 0.04
Siluridae	0.52 ± 0.02	0	0.52 ± 0.02	0.40 ± 0.02	0	0.40 ± 0.02
Clariidae	0	0	0	0.48 ± 0.04	0.22 ± 0.01	0.26 ± 0.02
Bagridae	0.54 ± 0.02	0.19 ± 0.01	0.35 ± 0.02	0.66 ± 0.02	0.37 ± 0.01	0.29 ± 0.03
Amblycipitidae	0.74 ± 0.01	0.25 ± 0.02	0.49 ± 0.02	0.80 ± 0.06	0.67 ± 0.01	0.13 ± 0.03
Sisoridae	0.63 ± 0.01	0	0.63 ± 0.01	0.90 ± 0.03	0	0.90 ± 0.03
Salangidae	0.72 ± 0.02	0.12 ± 0.02	0.60 ± 0.01	1.00	0	1.00
Hemiramphidae	0.38 ± 0.02	0	0.38 ± 0.02	0.80 ± 0.02	0	0.80 ± 0.02
Mastacembelidae	0.50 ± 0.03	0.17 ± 0.02	0.33 ± 0.02	0.68 ± 0.02	0.57 ± 0.01	0.11 ± 0.02
Eleotridae	0.20 ± 0.02	0	0.20 ± 0.02	0.54 ± 0.03	0	0.54 ± 0.03
Gobiidae	0.76 ± 0.02	0.34 ± 0.02	0.42 ± 0.01	0.56 ± 0.02	0	0.56 ± 0.02
Belontiidae	0	0	0	0.54	0	0.54
Channidae	0.50 ± 0.02	0	0.50 ± 0.02	0.33 ± 0.02	0	0.33 ± 0.02
Homalopteridae	0.81 ± 0.07	0.40 ± 0.04	0.41 ± 0.03	0.89 ± 0.06	0.33 ± 0.02	0.56 ± 0.05
Serranidae	0.26 ± 0.01	0.07 ± 0.02	0.19 ± 0.01	0.49 ± 0.03	0.17 ± 0.01	0.32 ± 0.02

Note

Values are the mean ± *SD*.

**Figure 4:**
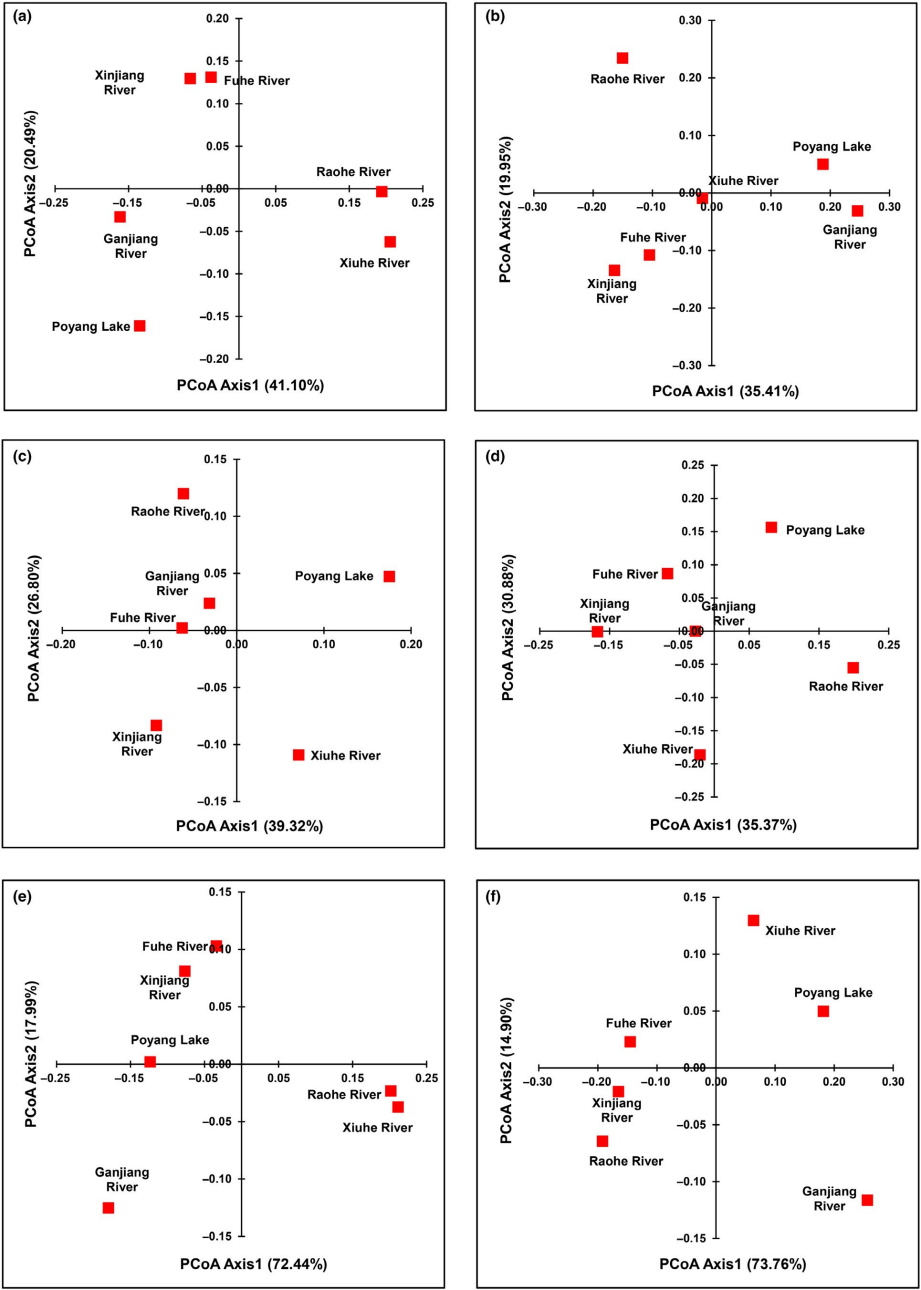
Results of the principal component analysis (PCoA) on the Sørensen index (*β*_sor_), and its spatial turnover component (*β*_sim_) and nestedness component (*β*_sne_) of the fish species in the historical (a, c, e) and current periods (b, d, f) in Poyang Lake Basin

The PCoA showed that the historical fish composition dissimilarity of the Xiuhe River and the Raohe River was similar, as was that of the Xinjiang River and the Fuhe River; the historical fish composition dissimilarity of Poyang Lake and the Ganjiang River was uniquely divided into other areas (Figure 4a,c,e). The current fish composition dissimilarity of the Xiuhe River, the Xinjiang River, and the Fuhe River was similar; the current fish composition dissimilarity of Poyang Lake, the Raohe River and the Ganjiang River was uniquely divided into other areas (Figure 4b,d,f).

We found a significant effect of the geographical distance on the spatial turnover component in the historical and current periods. In addition, we found the annual average runoff significantly affected the overall beta diversity in the current period, and the correlation between the nestedness component, the spatial turnover component, and the drainage area was significant in the current period (Table 4).

**Table 4:** Effects of geographical drivers on the pairwise Sørensen index (*β*_sor_) and its spatial turnover component (*β*_sim_) and nestedness component (*β*_sne_) for the two periods in Poyang Lake Basin, China

		Geographical distance		Annual average runoff		Drainage area	
		Historical	Current	Historical	Current	Historical	Current
*β*_sor_	*r*	−.029	.132	.292	.390	.047	.341
	*p*	.470	.250	.090	**.050** *	.510	.100
*β*_sim_	*r*	−.464	−.396	.267	−.219	−.523	−.522
	*p*	**.050** *	**.050** *	.160	.230	.100	**.040** *
*β*_sne_	*r*	.203	.313	.063	.335	.282	.516
	*p*	.230	.150	.160	.120	.120	**.050** *

Note

Significant results are in bold.

* *p* < .05.

## 4 DISCUSSION

### 4.1 Fish biodiversity declined

Historically, the Yangtze River formed a connected river–lake system (Fu et al., 2003; Jin et al., 2012; Zhang et al., 2013). Many fishes have developed a typical potamodromous life history, adapting to the seasonal flooding regime in this system (Cui & Li, 2005; Fu et al., 2003; Ren, He, Song, Cheng, & Xie, 2016). Therefore, river–lake connectivity and habitats are essential for migratory fish (Balcombe et al., 2007; Bayley, 1991; Fernandes, 1997; Osorio et al., 2011). Poyang Lake provides critical refuge for fish adapted to the river–floodplain system. Meanwhile, the migratory species of fish are more abundant in Poyang Lake Basin than in other areas (Kimura et al., 2012; Ren et al., 2016). However, the diversity of fish was declined over time in this basin (Huang et al., 2013). In this study, the numbers of fish species experienced significant decreases in Poyang Lake Basin; in particular, 36.7% of migratory fish were extirpated in the current period. Alpha and gamma diversity also experienced a significant decrease. Beta diversity clearly showed that there was an increase in the composition dissimilarity of the fish fauna in this basin. The PCoA also showed that the fish composition dissimilarity during the historical period was different from that of the current period.

### 4.2 Main threat

This study showed fish biodiversity was decreased in the current period, Poyang Lake Basin. The Chinese land areas had an area‐weighted average human footprint score of 7.93 in 2009 based on Venter et al. (2016a, 2016b), which is an increase of 9.4% from 1993 levels. In addition, land areas of Poyang Lake Basin had an area‐weighted average human footprint score of 16.22 in 2009, which is an increase of 14.3% from 1993 levels. The change of human footprint showed the human pressure increased over time, which accelerated the extirpation of some fish species (Dudgeon et al., 2006; Fu et al., 2003; Xie, 2017). Many factors, such as dam construction, sand mining, water pollution, overfishing, and species invasions, have been threatening fish biodiversity either directly or indirectly (Dudgeon et al., 2006; Raghavan, Prasad, Anvar‐Ali, & Pereira, 2008; Silvano, Hallwass, Juras, & Lopes, 2017; Trombulak & Frissell, 2000; Wu, Huang, Han, Xie, & Gao, 2003).

#### 4.2.1 Dam construction

Dam construction has been shown to have a far‐reaching impact on aquatic ecosystems (Liu, Hu, et al., 2017; Ming, Zhao, & Hui, 2004; Poff et al., 1997). Many basins have experienced habitat fragmentation and global loss caused by dams (Wozney, Haxton, Kjartanson, & Wilson, 2011; Wu et al., 2003), resulting in the decline in fish diversity (Daga et al., 2015; Petesse & Petrere, 2012; Vitule, Skóra, & Abilhoa, 2012). Indeed, the natural hydrological characteristics of Poyang Lake Basin were also changed by dams. There were 25 large reservoirs and 238 medium‐sized reservoirs in this basin. In this study, the beta diversity clearly showed that the increase in the composition dissimilarity of the fish fauna may be correlated with dam construction, particularly in the reservoir region above the dam.

Fish assemblages are the most sensitive population affected by dam construction and flow regulation (Larinier, 2000; Marchetti & Moyle, 2001; Wu et al., 2003). To adapt to natural flow regimes, fish have used different approaches, such as life history, and behavioral and morphological features (Lytle & Poff, 2004). In particular, migratory fish move upriver toward spawning grounds at the beginning of the rainy season, and the fish eggs then drift downriver while they develop and hatch in the turbid waters at the beginning of the flooding period (Agostinho, Gomes, Suzuki, & Júlio, 2003; Ren et al., 2016; Sá‐Oliveira, Hawes, Isaac‐Nahum, & Peres, 2015). For example, the construction of the Gezhouba Dam cut off the migration channel of *Acipenser sinensis* to their spawning grounds in the upper reach of the Yangtze River, resulting in the rapid decline of the population (Wei et al., 1997; Yu et al., 1988). Our study also showed 36.7% of migration fish were extirpated in the current period, such as *Acipenser sinensis*, *Tenualosa reevesii*, *Luciobrama macrocephalus*, and *Psephurus gladius*, which were not found in the current period. The population of *Coilia nasus* Temminck and Schlegel, 1846, *Myxocyprinus asiaticus* (Bleeker, 1864), and four major Chinese carps (*Mylopharyngodon piceus* [Richardson, 1846], *Ctenopharyngodon idella* [Valenciennes, 1844], *Hypophthalmichthys molitrix* [Valenciennes, 1844], and *Hypophthalmichthys nobilis* [Richardson, 1845]) declined rapidly. In addition, 37.7% of native species were extirpated per area in the current period. Many endemic species (*Tenualosa reevesii*, *Mylopharyngodon piceus*, *Ctenopharyngodon idella*) are replaced by cosmopolitan tolerant species (*Hemibarbus maculatus* [Bleeker, 1871]; *Squalidus argentatus* [Sauvage and Dabry de Thiersant, 1874]; *Pelteobagrus fulvidraco* [Richardson, 1846]; Allan, 2004; Chu et al., 2015).

#### 4.2.2 Sand mining

Sand mining causes the removal of sandbed resources (Hitchcock & Bell, 2004), changes of sediment composition (Cooper et al., 2007), water pollution (Hancock, 2002), a decline in the surface area of hydrophytes (Erftemeijer & Lewis, 2006), and a reduction in the richness of macrozoobenthos (Boyd, Limpenny, Rees, & Cooper, 2005), which is detrimental to the survival and reproduction of fish (Huang et al., 2013; Huang & Gong, 2007; Zhang & Huang, 2008; Zhong & Chen, 2005). Our study showed that 25% of carnivores, 18.7% of omnivores, and 23.8% of herbivores were extirpated, and 35.1% of demersal fish was extirpated in the current period. Indeed, many sand‐mining boats focused their extractions in this basin, which greatly blocked the spawning ground of fish, reducing the source of food (macrozoobenthos) for demersal and carnivores fish (such as *Myxocyprinus asiaticus*, *Mylopharyngododon piceus*), and affecting the reproduction of fish (such as *Cyprinus carpio* Linnaeus, 1758, *Carassius auratus* [Linnaeus, 1758]) that lay sticky eggs and attach them to hydrophytes (Hu, Hua, Zhou, Wu, & Wu, 2015; Huang et al., 2013; Liu, Hu, et al., 2017).

#### 4.2.3 Water pollution

Water pollution is also one of the main threats for the survival and reproduction of all fish (Dudgeon et al., 2006; Huang et al., 2013). With the development of industry and agriculture in Poyang Lake Basin, the continuous input of industrial wastewater and domestic sewage has caused the gradual deterioration of the water quality and indirectly affected community structure of fish (Huang et al., 2013; Wang et al., 2005; Zhang et al., 2011). This study showed that the composition dissimilarity of fish in the historical period was significantly different from that of the current period based on PCoA. Indeed, the water quality in this lake was relatively good in 1997–1999 (Zhang et al., 2012). It started deteriorating at the end of 20th century and is currently in transition to a state of eutrophication (Huang et al., 2013).

#### 4.2.4 Overfishing

Overfishing is one of the main threats for fish, which affected the community structure of fish, reduced the number of supplementary populations, and led to species becoming more threatened with extinction (Fulton, Smith, Smith, & Putten, 2011; Hilborn, 2007). This study also showed that the richness of fish species experienced significant decreases, and 27 fish species were listed as Critically Endangered (9), Endangered (3), Vulnerable (10), and Near Threatened (5) in the current period. For example, the yield of *Tenualosa reevesii* rapidly declined from 309–584 t in 1960, 74–157 t in 1970, and 12 t in 1986 due to overfishing (Fu et al., 2003; Huang et al., 2013; Liu, Chen, Duan, Qiu, & Wang, 2002). At the same time, a large number of fishing methods, such as traps, gill nets, and electrofishing, have been employed, leading to overfishing, which has also caused a dramatic decline in the fish biodiversity in this basin (Huang et al., 2013; Huang & Gong, 2007; Zhang, Wu, & Hu, 2010).

#### 4.2.5 Species invasions

Species invasion is also a serious threat to biodiversity (Frehse, Braga, Nocera, & Vitule, 2016; Pelicice, Vitule, Junior, Orsi, & Agostinho, 2014), which affects the survival of native fish species because they can compete with the native fishes for food, space, and other resources (Dudgeon & Smith, 2006; Raghavan et al., 2008; Welcomme & Vidthayanom, 2003). This study showed that beta diversity clearly showed that there was an increase in composition dissimilarity of introduced exotic species in the current period (0.68), which could increase the rate of extirpation of the native fish through competition or predation (Rahel, 2007). Indeed, species invasions have resulted in the significant impoverishment of Chinese freshwater fish fauna over the past century. For example, the number of native species in Dianchi Lake declined from 25 to 5 during 1940–2003 (Xie, Li, Gregg, & Li, 2001; Ye et al., 2015).

### 4.3 Conservation and management implications

Currently, information about some endangered species has helped to increase public awareness of the fish biodiversity in Poyang Lake Basin (Fu et al., 2003; Kang et al., 2014). Many measures have been taken to protect the fish biodiversity, but we believe that these efforts are still inadequate (Fu et al., 2003; Kang et al., 2014; Wu et al., 2003). Because Poyang Lake Basin is undergoing a very rapid deterioration as a result of human activities, conservation and management strategies must be improved and expanded. The following measures should be implemented to restore the fish resources in Poyang Lake Basin. First, nature reserves should be established in habitats rich in endemic species (Abell, Allan, & Lehner, 2007; Liu & Cao, 1992; Saunders, Meeuwig, & Vincent, 2002; Suski & Cooke, 2007). In this study, the nestedness component was the main contributor to beta diversity, which indicated one large protected area with a high species richness could be sufficient (Baiser, Olden, Record, Lockwood, & McKinney, 2012; Carvalho, Cardoso, & Gomes, 2012). Poyang Lake and the Ganjiang River Basin have high species richness and endemic fish, which indicates that more immediate conservation efforts should occur in these areas. Second, in global terms, Poyang Lake Basin is an area of interest for fish biodiversity, but it is also the most threatened basin, as shown by the rapidly declining fish populations. The conservation of fish requires further study on the complex life cycles and habitat requirements of fish. However, the life histories of only a few fish species have been studied. To this end, restoring fish resources should be a priority in Poyang Lake Basin, which provides a critical refuge for fish. Third, artificial propagation techniques are necessary to restore fish populations (Fu et al., 2003). To restore the fish populations, artificial propagation methods, such as restocking larvae and juveniles, should be used in Poyang Lake Basin. Finally, to establish and standardize the databases of fish biodiversity and habitat requirements, ecological networks should be established around Poyang Lake Basin that are associated with the biological laboratories of universities and museums (long‐term ecological research; Fu et al., 2003).

## CONFLICT OF INTEREST

The authors declare that there are no conflicts of interest.

## AUTHOR CONTRIBUTIONS

X. Liu, J. Qin, S. Ouyang and X. Wu conceived the study. All authors contributed to the study design and data collection. X. Liu and J. Qin analyzed the data. X. Liu, S. Ouyang and X. Wu led the writing of the manuscript.

## Data Availability

The data used in this manuscript were obtained from field investigations and laboratory experiments (taxon composition). We have attached the taxon information in supplemental files. Please see Tables [Link], [Link], [Link], [Link].

## References

[ece35661-bib-0001] Abell, R. , Allan, J. D. , & Lehner, B. (2007). Unlocking the potential of protected areas for freshwaters. Biological Conservation, 134, 48–63. 10.1016/j.biocon.2006.08.017

[ece35661-bib-0002] Agostinho, A. A. , Gomes, L. C. , Suzuki, I. S. , & Júlio, J. H. F. (2003). Migratory fishes of the Upper Paraná River Basin, Brazil. pp. 19–98 In Carolsfeld J., Harvey B., Ross C., & Baer A. (Eds.), Migratory fishes of South America: Biology, fisheries and conservation status (380 p). Victoria, BC: World Fisheries Trust.

[ece35661-bib-0003] Allan, J. D. (2004). Landscapes and riverscapes: The influence of land use on stream ecosystems. Annual Review of Ecology Evolution & Systematics, 35, 257–284. 10.1146/annurev.ecolsys.35.120202.110122

[ece35661-bib-0004] Arthington, A. H. , Dulvy, N. K. , Gladstone, W. , & Winfield, I. J. (2016). Fish conservation in freshwater and marine realms: Status, threats and management. Aquatic Conservation: Marine & Freshwater Ecosystems, 26, 838–857. 10.1002/aqc.2712

[ece35661-bib-0005] Baiser, B. , Olden, J. D. , Record, S. , Lockwood, J. L. , & McKinney, M. L. (2012). Pattern and process of biotic homogenization in the New Pangaea. Proceedings of the Royal Society B: Biological Sciences, 279, 4772–4777. 10.1098/rspb.2012.1651 PMC3497087 23055062

[ece35661-bib-0006] Balcombe, S. R. , Bunn, S. E. , Arthington, A. H. , Fawcett, J. H. , Mckenzie‐Smith, F. J. , & Wright, A. (2007). Fish larvae, growth and biomass relationships in an Australian arid zone river: Links between floodplains and waterholes. Freshwater Biology, 52, 2385–2398. 10.1111/j.1365-2427.2007.01855.x

[ece35661-bib-0007] Baselga, A. (2010). Partitioning the turnover and nestedness components of beta diversity. Global Ecology & Biogeography, 19, 134–143. 10.1111/j.1466-8238.2009.00490.x

[ece35661-bib-0008] Baselga, A. , & Orme, C. D. L. (2012). betapart: An R package for the study of beta diversity. Methods in Ecology & Evolution, 3, 808–812.

[ece35661-bib-0009] Bayley, P. B. (1991). The flood pulse advantage and the restoration of river–floodplain systems. Regulated Rivers: Research & Management, 6, 75–86. 10.1002/rrr.3450060203

[ece35661-bib-0010] Bergamin, R. S. , Bastazini, V. A. G. , Vélez‐Martin, E. , Debastiani, V. , Zanini, K. , Loyola, R. , & Müller, S. C. (2017). Linking beta diversity patterns to protected areas: Lessons from the Brazilian Atlantic Rainforest. Biodiversity & Conservation, 26, 1–12. 10.1007/s10531-017-1315-y

[ece35661-bib-0011] Boyd, S. E. , Limpenny, D. S. , Rees, H. L. , & Cooper, K. M. (2005). The effects of marine sand and gravel extraction on the macrobenthos at a commercial dredging site (results 6 years post‐dredging). ICES Journal of Marine Science, 62, 145–162. 10.1016/j.icesjms.2004.11.014

[ece35661-bib-0012] Cardinale, B. J. , Duffy, J. E. , Gonzalez, A. , Hooper, D. U. , Perrings, C. , Venail, P. , … Naeem, S. (2012). Biodiversity loss and its impact on humanity. Nature, 489, 326 10.1038/nature11148 22678280

[ece35661-bib-0013] Carvalho, J. C. , Cardoso, P. , & Gomes, P. (2012). Determining the relative roles of species replacement and species richness differences in generating beta‐diversity patterns. Global Ecology & Biogeography, 21, 760–771. 10.1111/j.1466-8238.2011.00694.x

[ece35661-bib-0014] Chao, A. , Ma, K. H. , & Hsieh, T. C. (2016). iNEXT (iNterpolation and EX Trapolation) Online: Software for Interpolation and Extrapolation of Species Diversity. Program and User's Guide. Retrieved from http://chao.stat.nthu.edu.tw/wordpress/software_download/

[ece35661-bib-0015] Chu, L. , Wang, W. J. , Zhu, R. , Yan, Y. Z. , Chen, Y. F. , & Wang, L. Z. (2015). Variation in fish assemblages across impoundments of low‐head dams in headwater streams of the Qingyi River, China: Effects of abiotic factors and native invaders. Environmental Biology of Fishes, 98, 101–112. 10.1007/s10641-014-0239-6

[ece35661-bib-0016] Cooper, K. , Boyd, S. , Eggleton, J. , Limpenny, D. , Rees, H. , & Vanstaen, K. (2007). Recovery of the seabed following marine aggregate dredging on the Hastings Shingle Bank off the southeast coast of England. Estuarine Coastal & Shelf Science, 75, 547–558. 10.1016/j.ecss.2007.06.004

[ece35661-bib-0017] Cressey, D. (2009). Aquaculture: Future fish. Nature, 458, 398–400. 10.1038/458398a 19325603

[ece35661-bib-0018] Cui, Y. B. , & Li, Z. J. (2005). Fishery resources and conservation of environment in Lakes of the Changjiang River Basin (pp. 181–192). Beijing, China: Science Press.

[ece35661-bib-0019] Daga, V. D. , Skóra, F. , Padial, A. A. , Abilhoa, V. , Gubiani, E. A. , & Vitule, J. R. S. (2015). Homogenization dynamics of the fish assemblages in Neotropical reservoirs: Comparing the roles of introduced species and their vectors. Hydrobiologia, 746, 327–347. 10.1007/s10750-014-2032-0

[ece35661-bib-0020] De Silva, S. S. (2012). Aquaculture: A newly emergent food production sector and perspectives of its impacts on biodiversity and conservation. Biodiversity & Conservation, 21, 3187–3220. 10.1007/s10531-012-0360-9

[ece35661-bib-0021] Dray, S. , & Dufour, A. (2007). The ade4 package: Implementing the duality diagram for ecologists. Journal of Statistical Software, 22, 1–20.

[ece35661-bib-0022] Dudgeon, D. , Arthington, A. H. , Gessner, M. O. , Kawabata, Z.‐I. , Knowler, D. J. , Lévêque, C. , … Sullivan, C. A. (2006). Freshwater biodiversity: Importance, threats, status, and conservation challenges. Biological Reviews, 81, 163–182. 10.1017/S1464793105006950 16336747

[ece35661-bib-0023] Dudgeon, D. , & Smith, R. E. W. (2006). Exotic species, fisheries and conservation of freshwater biodiversity in tropical Asia: The case of the Sepik River, Papua New Guinea. Aquatic Conservation: Marine & Freshwater Ecosystem, 16, 203–215. 10.1002/aqc.713

[ece35661-bib-0024] Erftemeijer, P. L. A. I. , & Lewis, R. R. R. (2006). Environmental impacts of dredging on seagrasses: A review. Marine Pollution Bulletin, 52, 1553–1572. 10.1016/j.marpolbul.2006.09.006 17078974

[ece35661-bib-0025] Fernandes, C. C. (1997). Lateral migration of fishes in Amazon floodplains. Ecology of Freshwater Fish, 6, 36–44. 10.1111/j.1600-0633.1997.tb00140.x

[ece35661-bib-0026] Frehse, F. A. , Braga, R. R. , Nocera, G. A. , & Vitule, J. R. S. (2016). Non‐native species and invasion biology in a megadiverse country: Scientometric analysis and ecological interactions in Brazil. Biological Invasions, 18, 3713–3725. 10.1007/s10530-016-1260-9

[ece35661-bib-0027] Fu, C. , Wu, J. , Chen, J. , Wu, Q. , & Lei, G. (2003). Freshwater fish biodiversity in the Yangtze River basin of China: Patterns, threats and conservation. Biodiversity & Conservation, 12, 1649–1685.

[ece35661-bib-0028] Fulton, E. A. , Smith, A. D. M. , Smith, D. C. , & van Putten, I. E. (2011). Human behaviour: The key source of uncertainty in fisheries management. Fish & Fisheries, 12, 2–17. 10.1111/j.1467-2979.2010.00371.x

[ece35661-bib-0029] Hancock, P. J. (2002). Human impacts on the stream‐groundwater exchange zone. Environmental Management, 29, 763–781. 10.1007/s00267-001-0064-5 11992170

[ece35661-bib-0030] Hilborn, R. (2007). Managing fisheries is managing people: What has been learned? Fish & Fisheries, 8, 285–296. 10.1111/j.1467-2979.2007.00263_2.x

[ece35661-bib-0031] Hitchcock, D. R. , & Bell, S. (2004). Physical impacts of marine aggregate dredging on seabed resources in coastal deposits. Journal of Coastal Research, 20, 101–114. 10.2112/1551-5036(2004)20[101:PIOMAD]2.0.CO;2

[ece35661-bib-0032] Hu, M. , Hua, Q. , Zhou, H. , Wu, Z. , & Wu, X. (2015). The effect of dams on the larval abundance and composition of four carp species in key river systems in china. Environmental Biology of Fishes, 98, 1201–1205. 10.1007/s10641-014-0342-8

[ece35661-bib-0033] Huang, L. L. , Wu, Z. Q. , & Li, J. H. (2013). Fish fauna, biogeography and conservation of freshwater fish in Poyang Lake Basin, China. Environmental Biology of Fishes, 96, 1229–1243. 10.1007/s10641-011-9806-2

[ece35661-bib-0034] Huang, X. P. , & Gong, Y. (2007). Fishery resources in Poyang Lake and its conservation. Jiangxi Fishery Science Technology, 112, 2–6.

[ece35661-bib-0035] Jiang, Z. G. , Jiang, J. P. , Wang, Y. Z. , Zhang, E. , Zhang, Y. Y. , … Ping, X. G. (2016). Red list of China's vertebrates. Biodiversity Science, 24, 500–551.

[ece35661-bib-0036] Jin, B. S. , Nie, M. , Li, Q. , Chen, J. K. , & Zhou, W. B. (2012). Basic characteristics, challenges and key scientific questions of the Poyang Lake basin. Resources and Environment in the Yangtze Basin, 21, 268–275.

[ece35661-bib-0037] Kang, B. , Deng, J. M. , Wu, Y. F. , Chen, L. Q. , Zhang, J. , Qiu, H. Y. , … He, D. M. (2014). Mapping China's freshwater fishes: Diversity and biogeography. Fish and Fisheries, 15, 209–230. 10.1111/faf.12011

[ece35661-bib-0038] Kimura, S. , Akamatsu, T. , Li, S. H. , Dong, L. J. , Wang, K. X. , Wang, D. , & Arai, N. (2012). Seasonal changes in the local distribution of Yangtze finless porpoises related to fish presence. Marine Mammal Science, 28, 308–324. 10.1111/j.1748-7692.2011.00490.x

[ece35661-bib-0039] Larinier, M. (2000). Dams and fish migration, Institut de Mecanique des Fluides, Toulouse, France. Prepared for thematic review II. 1: Dams, ecosystem functions and environmental restoration.

[ece35661-bib-0040] Legendre, P. , & De Cáceres, M. (2013). Beta diversity as the variance of community data: Dissimilarity coefficients and partitioning. Ecology Letters, 16, 951–963. 10.1111/ele.12141 23809147

[ece35661-bib-0041] Legendre, P. , & Legendre, L. (2012). Numerical ecology (3rd ed.). Amsterdam, The Netherlands: Elsevier.

[ece35661-bib-0042] Liu, S. P. , Chen, D. Q. , Duan, X. B. , Qiu, S. L. , & Wang, L. M. (2002). The resources status quo and protection strategies on Chinese Shad. Acta Hydrobiologica Sinica, 26, 679–684.

[ece35661-bib-0043] Liu, J. , & Cao, W. (1992). Fish resources of the Yangtze River basin and the tactics for their conservation. Resources and Environment in the Yangtze Valley, 1, 17–23.

[ece35661-bib-0044] Liu, X. J. , Hu, X. Y. , Ao, X. F. , Wu, X. P. , & Ouyang, S. (2017). Community characteristics of aquatic organisms and management implications after construction of Shihutang Dam in the Gangjiang River, China. Lake and Reservoir Management, 34, 42–57. 10.1002/9780470015902.a0020471.pub2

[ece35661-bib-0045] Lytle, D. A. , & Poff, N. L. (2004). Adaption to natural flow regimes. Trends in Ecology & Evolution, 19, 94–100. 16701235 10.1016/j.tree.2003.10.002

[ece35661-bib-0046] Marchetti, M. P. , & Moyle, P. B. (2001). Effects of flow regime on fish assemblages in a regulated California stream. Ecological Applications, 11, 530–539. 10.1890/1051-0761(2001)011[0530:EOFROF]2.0.CO;2 22908707

[ece35661-bib-0047] Mcknight, M. W. , White, P. S. , Mcdonald, R. I. , Lamoreux, J. , Sechrest, W. , Ridgely, R. S. , & Stuart, S. N. (2007). Putting beta‐diversity on the map: Broad‐scale congruence and coincidence in the extremes. PLoS Biology, 5, e272 10.1371/journal.pbio.0050272 17927449 PMC2001212

[ece35661-bib-0048] Ming, H. D. , Zhao, W. J. , & Hui, C. L. (2004). The ecological changes in manwan reservoir area and its causes. Journal of Yunnan University, 26, 220–226.

[ece35661-bib-0049] Monnet, A. C. , Jiguet, F. , Meynard, C. N. , Mouillot, D. , Mouquet, N. , Thuiller, W. , & Devictor, V. (2014). Asynchrony of taxonomic, functional and phylogenetic diversity in birds. Global Ecology & Biogeography, 23, 780–788. 10.1111/geb.12179 25067904 PMC4110699

[ece35661-bib-0050] Naylor, R. L. , Goldburg, R. J. , Primavera, J. H. , Kautsky, N. , Beveridge, M. C. M. , Clay, J. , … Troell, M. (2000). Effect of aquaculture on world fish supplies. Nature, 405, 1017–1024. 10.1038/35016500 10890435

[ece35661-bib-0051] Nogueira, C. , Buckup, P. A. , Menezes, N. A. , Oyakawa, O. T. , Kasecker, T. P. , Mario, B. , & Da Silva, J. M. C. (2010). Restricted‐range fishes and the conservation of Brazilian freshwaters. PLoS ONE, 5, e11390 10.1371/journal.pone.0011390 20613986 PMC2894945

[ece35661-bib-0052] Oksanen, J. , Blanchet, F. G. , Kindt, R. , Legendre, P. , Minchin, P. R. , O'Hara, R. , … Wagner, H. (2015). vegan: community ecology package. R package version 2.3‐2. Retrieved from http://cran.r-project.org

[ece35661-bib-0053] Olden, J. D. , Comte, L. , & Giam, X. (2016). Biotic homogenisation In Encyclopedia of Life Sciences (ELS) (pp. 1–8). Chichester, UK: John Wiley & Sons 10.1002/9780470015902.a0020471.pub2

[ece35661-bib-0054] Olden, J. D. , & Rooney, T. P. (2006). On defining and quantifying biotic homogenization. Global Ecology & Biogeography, 15, 113–120. 10.1111/j.1466-822X.2006.00214.x

[ece35661-bib-0055] Osorio, D. , Terborgh, J. , Alvarez, A. , Ortega, H. , Quispe, R. , Chipollini, V. , & Davenport, L. C. (2011). Lateral migration of fish between an oxbow lake and an Amazonian headwater river. Ecology of Freshwater Fish, 20, 619–627. 10.1111/j.1600-0633.2011.00511.x

[ece35661-bib-0056] Pelicice, F. M. , Vitule, J. R. S. , Junior, L. A. , Orsi, M. L. , & Agostinho, A. A. (2014). A serious new threat to Brazilian freshwater ecosystems: The naturalization of nonnative fish by decree. Conservation Letters, 7, 55–60. 10.1111/conl.12029

[ece35661-bib-0057] Petesse, M. L. , & Petrere, J. M. (2012). Tendency towards homogenization in fish assemblages in the cascade reservoir system of the Tietê River basin, Brazil. Ecological Engineering, 48, 109–116. 10.1016/j.ecoleng.2011.06.033

[ece35661-bib-0058] Poff, N. L. , Allan, J. D. , Bain, M. B. , Karr, J. R. , Prestegaard, K. L. , Richter, B. D. , … Stromberg, J. C. (1997). The natural flow regime. BioScience, 47, 769–784. 10.2307/1313099

[ece35661-bib-0059] Qian, H. , & Ricklefs, R. E. (2006). The role of exotic species in homogenizing the North American flora. Ecology Letters, 9, 1293–1298. 10.1111/j.1461-0248.2006.00982.x 17118003

[ece35661-bib-0060] R Development Core Team (2014). R: A language and environment for statistical computing. Vienna, Austria: R Foundation for Statistical Computing Retrieved from http://Rproject.org

[ece35661-bib-0061] Raghavan, R. , Prasad, G. , Anvar‐Ali, P. H. , & Pereira, B. (2008). Exotic fish species in a global biodiversity hotspot: Observations from River Chalakudy, part of Western Ghats, Kerala, India. Biological Invasions, 10, 37–40. 10.1007/s10530-007-9104-2

[ece35661-bib-0062] Rahel, F. J. (2007). Biogeographic barriers, connectivity and homogenization of freshwater faunas: It's a small world after all. Freshwater Biology, 52, 696–710. 10.1111/j.1365-2427.2006.01708.x

[ece35661-bib-0063] Ren, P. , He, H. , Song, Y. Q. , Cheng, F. , & Xie, S. G. (2016). The spatial pattern of larval fish assemblages in the lower reach of the Yangtze River: Potential influences of river–lake connectivity and tidal intrusion. Hydrobiologia, 766, 365–379. 10.1007/s10750-015-2471-2

[ece35661-bib-0064] Sá‐Oliveira, J. C. , Hawes, J. E. , Isaac‐Nahum, V. J. , & Peres, C. A. (2015). Upstream and downstream responses of fish assemblages to an eastern amazonian hydroelectric dam. Freshwater Biology, 60, 2037–2050. 10.1111/fwb.12628

[ece35661-bib-0065] Saunders, D. L. , Meeuwig, J. J. , & Vincent, A. C. J. (2002). Freshwater protected areas: Strategies for conservation. Conservation Biology, 16, 30–41. 10.1046/j.1523-1739.2002.99562.x 35701954

[ece35661-bib-0066] Silvano, R. A. M. , Hallwass, G. , Juras, A. A. , & Lopes, P. F. M. (2017). Assessment of efficiency and impacts of gillnets on fish conservation in a tropical freshwater fishery. Aquatic Conservation: Marine & Freshwater Ecosystems, 27, 521–533. 10.1002/aqc.2687

[ece35661-bib-0067] Suski, C. D. , & Cooke, S. J. (2007). Conservation of aquatic resources through the use of freshwater protected areas: Opportunities and challenges. Biodiversity & Conservation, 16, 2015–2029. 10.1007/s10531-006-9060-7

[ece35661-bib-0068] Taylor, E. B. (2010). Changes in taxonomy and species distributions and their influence on estimates of faunal homogenization and differentiation in freshwater fishes. Diversity & Distributions, 16, 676–689. 10.1111/j.1472-4642.2010.00670.x

[ece35661-bib-0069] Toussaint, A. , Beauchard, O. , Oberdorff, T. , Brosse, S. , & Villeger, S. (2014). Historical assemblage distinctiveness and the introduction of widespread non‐native species explain worldwide changes in freshwater fish taxonomic dissimilarity. Global Ecology & Biogeography, 23, 574–584. 10.1111/geb.12141

[ece35661-bib-0070] Trombulak, S. C. , & Frissell, C. A. (2000). Review of ecological effects of roads on terrestrial and aquatic communities. Conservation Biology, 14, 18–30. 10.1046/j.1523-1739.2000.99084.x

[ece35661-bib-0071] Venter, O. , Sanderson, E. W. , Magrach, A. , Allan, J. R. , Beher, J. , Jones, K. R. , … Watson, J. E. M. (2016a). Data descriptor: Global terrestrial Human Footprint maps for 1993 and 2009. Scientific Data, 3, 160067 10.1038/sdata.2016.67 27552448 PMC5127486

[ece35661-bib-0072] Venter, O. , Sanderson, E. W. , Magrach, A. , Allan, J. R. , Beher, J. , Jones, K. R. , … Watson, J. E. M. (2016b). Sixteen years of change in the global terrestrial human footprint and implications for biodiversity conservation. Nature Communications, 7, 12558 10.1038/ncomms12558 PMC4996975 27552116

[ece35661-bib-0073] Vitule, J. R. S. , Skóra, F. , & Abilhoa, V. (2012). Homogenization of freshwater fish faunas after the elimination of a natural barrier by a dam in neotropics. Diversity & Distributions, 18, 111–120. 10.1111/j.1472-4642.2011.00821.x

[ece35661-bib-0074] Wang, K. , Shi, X. Z. , Yu, D. S. , Shi, D. M. , Chen, J. M. , Xu, B. B. , … Li, D. C. (2005). Environmental factors affecting temporal and spatial dynamics of soil erosion in Xingguo County, South China. Pedosphere, 15, 620–627.

[ece35661-bib-0075] Wei, Q. , Ke, F. , Zhang, J. , Zhuang, P. , Luo, J. , Zhou, R. , & Yang, W. H. (1997). Biology, fisheries, and conservation of sturgeons and paddlefish in china. Environmental Biology of Fishes, 48, 241–255.

[ece35661-bib-0076] Welcomme, R. , & Vidthayanom, C. (2003). The impacts of introductions and stocking of exotic species in the Mekong Basin and policies for their control. MRC Technical Paper No. 9, Mekong River Commission, Phnom Penh.

[ece35661-bib-0077] Whittaker, R. H. (1960). Vegetation of the Siskiyou Mountains, Oregon and California. Ecological Monographs, 30, 279–338. 10.2307/1943563

[ece35661-bib-0078] Wiersma, Y. F. , & Urban, D. L. (2005). Beta diversity and nature reserve system design in the Yukon, Canada. Conservation Biology, 19, 1262–1272. 10.1111/j.1523-1739.2005.00099.x

[ece35661-bib-0079] Winter, M. , Schweiger, O. , Klotz, S. , Nentwig, W. , Andriopoulos, P. , Arianoutsou, M. , … Kuhn, I. (2009). Plant extinctions and introductions lead to phylogenetic and taxonomic homogenization of the European flora. Proceedings of the National Academy of Sciences of the United States of America, 106, 21721–21725. 20007367 10.1073/pnas.0907088106 PMC2792159

[ece35661-bib-0080] Wozney, K. M. , Haxton, T. J. , Kjartanson, S. , & Wilson, C. C. (2011). Genetic assessment of lake sturgeon (*Acipenser fulvescens*) population structure in the Ottawa River. Environmental Biology of Fishes, 90, 183–195. 10.1007/s10641-010-9730-x

[ece35661-bib-0081] Wu, J. , Huang, J. , Han, X. , Xie, Z. , & Gao, X. (2003). Three‐Gorges Dam‐experiment in habitat fragmentation? Science, 300, 1239–1240. 12764179 10.1126/science.1083312

[ece35661-bib-0082] Xie, Y. , Li, Z. , Gregg, W. P. , & Li, D. (2001). Invasive species in China – An overview. Biodiversity and Conservation, 10, 1317–1341.

[ece35661-bib-0083] Xie, P. (2017). Biodiversity crisis in the Yangtze River: the culprit was dams, followed by overfishing. Journal of Lake Science, 29, 1279–1299.

[ece35661-bib-0084] Yan, Y. Z. , Xiang, X. Y. , Chu, L. , Zhan, Y. J. , & Fu, C. Z. (2011). Influences of local habitat and stream spatial position on fish assemblages in a dammed watershed, the Qingyi stream, China. Ecology of Freshwater Fish, 20, 199–208. 10.1111/j.1600-0633.2010.00478.x

[ece35661-bib-0085] Ye, S. W. , Lin, M. L. , Lin, L. , Liu, J. S. , Song, L. R. , & Li, Z. J. (2015). Abundance and spatial variability of invasive fishes related to environmental factors in a eutrophic Yunnan Plateau lake, Lake Dianchi, Southwestern China. Environmental Biology of Fishes, 98, 209–224. 10.1007/s10641-014-0252-9

[ece35661-bib-0086] Ye, F. L. , & Zhang, J. D. (2002). Fish ecology. Guangzhou, China: Guangdong Higher Education Press.

[ece35661-bib-0087] Yu, Z. , Deng, Z. , Xu, Y. , Cai, M. , Zhao, Y. , Liang, Z. , … Zeng, X. (1988). The present situation of the spawning grounds of the four Chinese domestic fishes in the Yangtze River after the construction of the Gezhouba Water control project In Yi B., Yu Z., & Liang Z. (Eds.), Gezhouba Water control project and four domestic fishes in Yangtze River (pp. 47–68). Wuhan, China: Hubei Science and Technology Press.

[ece35661-bib-0088] Zhang, G. , Wu, L. , Li, H. T. , Liu, M. , Cheng, F. , Murphy, B. R. , & Xie, S. G. (2012). Preliminary evidence of delayed spawning and suppressed larval growth and condition of the major carps in the Yangtze River below the Three Gorges Dam. Environmental Biology of Fishes, 93, 439–447. 10.1007/s10641-011-9934-8

[ece35661-bib-0089] Zhang, J. M. , Wu, Z. Q. , & Hu, M. L. (2010). Resource status of four major Chinese carps in the Xiajiang reach of Ganjiang River. Journal of Hydroecology, 3, 34–37.

[ece35661-bib-0090] Zhang, M. H. , Xu, L. , Xie, G. L. , Liu, Y. B. , Liu, X. M. , Song, S. C. , … Wu, X. P. (2013). Species diversity, distribution and conservation of freshwater mollusk in Poyang Lake basin. Marine Sciences, 37, 114–124.

[ece35661-bib-0091] Zhang, Z. L. , & Huang, L. Z. (2008). Influence of quarrying in Poyang Lake in the ecological environment. Jiangxi Hydraulic Science Technology, 34, 7–10.

[ece35661-bib-0092] Zhang, Z. Y. , Liu, M. , Peng, A. C. , Hu, L. , Wang, C. Y. , & Chen, W. X. (2011). Main problems and restoration measures of fish habitat in Poyang Lake. Journal of Hydroecology, 32, 134–136.

[ece35661-bib-0093] Zhong, Y. X. , & Chen, S. (2005). Effect of sand mining on fish in Poyang Lake. Jiangxi Fishery Science & Technology, 1, 15–18.

