# Supplementary figures and images for: Biodiversity pattern of fish assemblages in Poyang Lake Basin: Threat and conservation
Source: Ecol Evol. 2019 Sep 26;9(20):11672–83. doi: 10.1002/ece3.5661 (PMC6822132; doi:10.1002/ece3.5661)

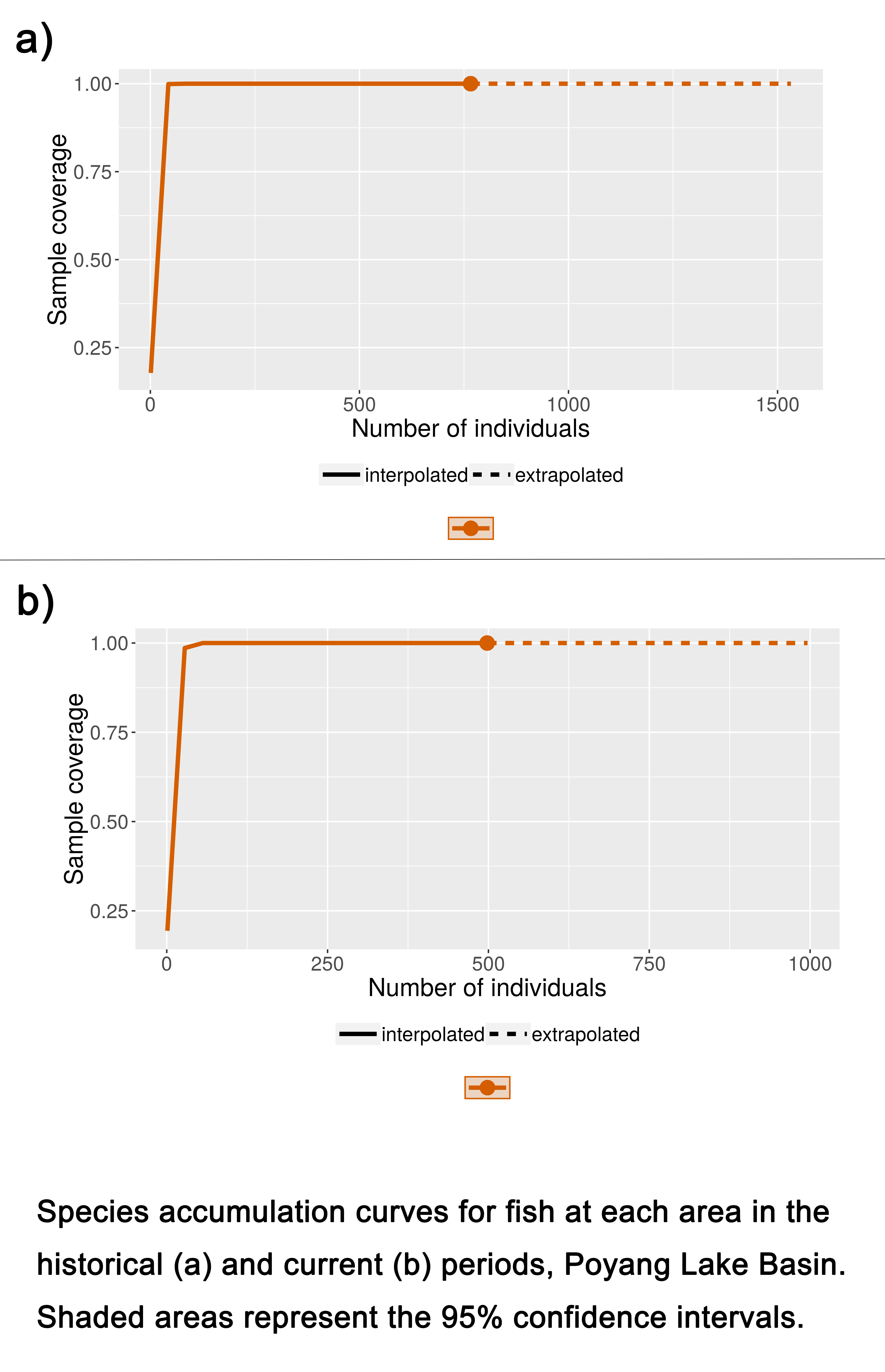

Supplement: Supplementary file 1 [file ECE3-9-11672-s001.tif]

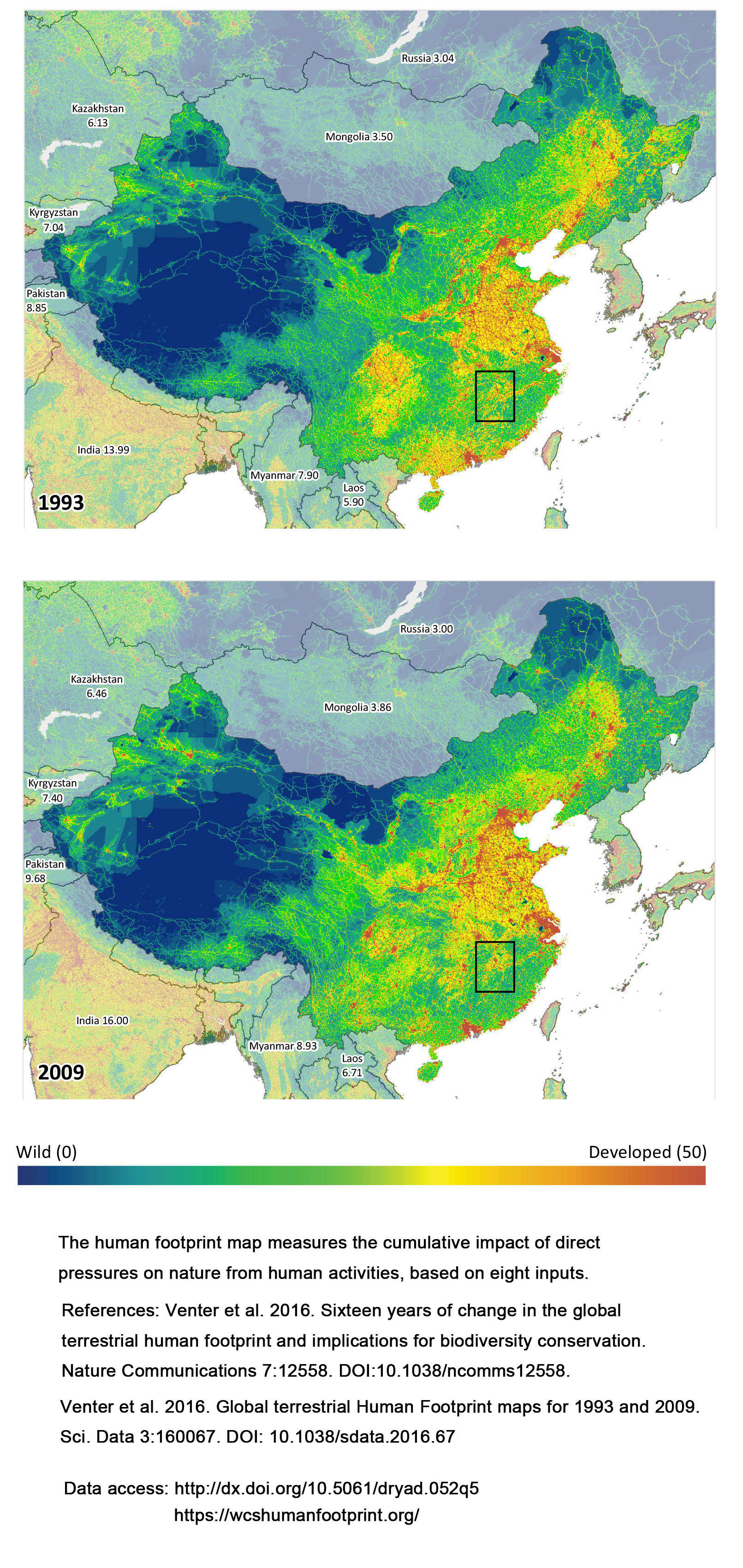

Supplement: Supplementary file 2 [file ECE3-9-11672-s002.tif]
