# Supplementary material for: Biodiversity pattern of fish assemblages in Poyang Lake Basin: Threat and conservation
Source: Ecol Evol. 2019 Sep 26;9(20):11672–83. doi: 10.1002/ece3.5661 (PMC6822132; doi:10.1002/ece3.5661)
Supplement: Supplementary file 3 [file ECE3-9-11672-s003.docx]

**Table S1** Literature used to buildup fish species lists in the historical (1980-2000), and the current (2000-2017) periods in Poyang Lake Basin.

| Basins | Historical | Current |
| --- | --- | --- |
| Poyang Lake | 1;2;3;4; | 5;6;7;8;9;10;11;12;13;14 |
| Ganjiang River | 1;2;15;16;17;18 | 19;20;21;22;23;24;25;26;27;28;12;13;14 |
| Fuhe River | 1;2;29;30 | 31;32;12;13;14 |
| Xinjiang River | 1;2;33 | 34;35;36;12;13;14 |
| Raohe River | 1;2 | 37;12;13;14 |
| Xiuhe River | 1;2 | 38;39;40;12;13;14 |

**References**

1. Jiang, Y.J. (1985) Preliminary analysis of fish fauna in Jiangxi province. *Jiangxi Fishery Sciences and Technology*, 1, 1-16.
2. Guo, Z.Z.＆Liu, R.L. (1995) The fishes of Jiangxi Province. *Journal of Nanchang University*, 19, 222-232.
3. Cui, Y.B.＆Li, Z.J. (2005) Fishery resources and conservation of environment in lakes of the Changjiang River basin. Science Press, Beijing.
4. Zhang, T.L.＆Li, Z.J. (2007) Fish resources and fishery utilization of Poyang Lake. *Journal of Lake Sciences*, 19, 434-444.
5. Hu, M., Wu, Z.Q.＆Zhou, M.H. (2005) The fisheries characters and resource status of Nanjishan Natural Reserve in Poyang Lake. *Resources and Environmental in the Yangtze Basin*, 14, 561-565.
6. Hu, M., Wu, Z.Q.＆Liu, Y.L. (2011) Fish diversity and community structure in Hukou area of Lake Poyang. *Journal of Lake Sciences*, 23, 246-250.
7. Zeng. Z., Zhang, X.C.＆Liu, G.H. (2015) Structure and features of fishery resources of sub-lakes in Poyang Lake, Jiangxi, China. *Resources and Environment in the Yangtze Basin*, 24, 1021-1029.
8. Yang, S., LI, M.Z.＆ZHU, Q.G. (2015) Spatial and temporal variations of fish assemblages in Poyang Lake. *Resources and Environment in the Yangtze Basin*, 24, 54–64.
9. He, G, Fang, C.L.＆Chen, W.J. (2015) Community structure and variation of migration fishes in Pingfeng area of Poyang Lake channel. *Hubei Agricultural Sciences*, 54, 926-930.
10. Fang, C., Chen, W.J.＆Zhou, H.M. (2016) Fish resources in Poyang Lake and their utilization. *Jiangsu Agricultural Sciences*, 44, 233-243.
11. Wang, S., Duan, X.B.＆Chen, W.J. (2016) Status and changes of fish resources in the Hukou area of Poyang Lake. *Freshwater Fisheries*, 46, 50-56.
12. Huang, G.S. (2014) The Biological Invasions and Quarantine Inspection in China. China Zhijian Publishing House, Beijing.
13. Xu, H.G.＆Qiang, S. (2011) China’s Invasive Alien Species. Science Press, Beijing.
14. Wang, R., Fu, Q.W.＆Liu, J.L. (2014) Atlas of main Aquatic products in Poyang Lake, Jiangxi Science and Technology Press, Nanchang.
15. Zou, D.L. (1982) Fishery resources in Jiulianshan area of Jiangxi Province. *Journal of Nanchang University*, 2, 50-53.
16. Tian, J.L. (1989) Fishery Utilization in Ganjiang River before Wanan Water Control Project. *Freshwater Fisheries*, 1, 33-39.
17. Zhang, E. Liu, H.Z.＆He, C.C. (1996) The fish fauna in northeast Jiangxi. *Chinese Journal of zoology*, 31, 3-12.
18. Wang, X.，Luo, T.H.＆Zhou. H.Z. (2005) Large-scale patterns in species diversity of fishes in the Yangtze River Basin. *Biodiversity Science*, 13, 473-495.
19. Li, Q, Wu, Z.Q.＆Huang, L.L. (2008) Fish resources of Qiyunshan Natural Reserve, Jiangxi, China. *Acta Zootaxonomica Sinica*, 32, 324-329.
20. Zhang, J., Wu, Z.Q.＆Hu, M.L. (2009) Status of fish resource in Xiajiang reach of middle Ganjiang River. *Jiangxi Science*, 27, 916-919.
21. Zou, S., Wu, Z.Q.＆Hu, M.L. (2010) The predicative analysis about the effects of Xiajiang water control project on fish resource in the Middle Reaches of Gan River. *Journal of* *Nanchang University*, 34, 290-293.
22. Hu, M., Wu, Z.Q.＆Liu, Y.L. (2010) Fish resources status of Taihe section in middle reaches of Gan River. *Journal of Nanchang University*, 34, 90-93.
23. Zou, S., Wu, Z.Q.＆Hu, M.L. (2010) Effect of Shihutang Navigation and Hydropower Project in Ganjiang River on Fish Resource. *Journal of Guilin University of Technology*, 30, 268-271.
24. Chen, H., Zhang, M.＆Liu, Z.G. (2011) Assessment of freshwater ecosystem integrity and health in Ganjiang River basin through the fish IBI method. *Resources and Environment in the Yangtze Basin*, 20, 1098-1107.
25. Hu, M.L. Wu, Z.Q.＆Li, Q. (2011) Preliminary research on fish species diversity of Ganjiangyuan Nature Reserve in Jiangxi Province. *Sichuan Journal of Zoology*, 30,467-470.
26. Su, N, Xiao, D, Li, l.＆Xu, Z.Q. (2012) Status of fish resources in Xiajiang to Nanchang section of Ganjiang River. *Journal of Huazhong Agricultural University*, 31, 756-764.
27. Guo, S. (2012) Fish species diversity status of Taiji section in middle reaches of Gan River. *Nanchang University*.
28. Miao, C, Zhu, Y.H.＆Yuan, R.B. (2016) Fish species diversity in Jinpenshan Nature Reserve Zone, Jiangxi Province, in summer. *Chinese journal of fisheries*, 29, 28-32.
29. Liu, S.P. (1985) The investigation on fish resources of Fu-He valley in Jiangxi. *Journal of Jiangxi University*, 1, 68-71.
30. Fu, J., Huang, S.L.＆Tong, S.M. (1996) Investigation report on fish resources in Fuzhou area. *Jiangxi Fishery Sciences and Technology*, 2, 5-9.
31. Hua, Q. (2010) Genetic diversity and resources of the four major Chinese carps in the Fuzhou reaches of the Fu River. *Nanchang University*.
32. Hu, M., Xi, H.B.＆Hua, Q. (2014) Preliminary investigations on fish resources of Yihuang *Mergus squamatus* Natural Reserve in Jiangxi. *Journal of Nanchang University*, 38, 502-505.
33. Guo, Z.Z.＆Liu, R.L. (1983) Investigation on fish resources of Yujiang County (Xin River), Jiangxi Province. *Journal of Jiangxi University*, 1, 11-21.
34. Chen, Y.L. (2010) Genetic diversity and resources of the four major Chinese carps in the YingTan reaches of the Xin River. *Nanchang University*.
35. Guo, S. Wu, Z.Q.＆Hu, M.L. (2011) Preliminary survey of fish in Yangjifeng Nature Reserve, Jiangxi Province. *Journal of Hydroecology*, 32, 142-144.
36. Lu, M., Jiang, T.＆Liu, H.B. (2015) Existence of anadromous *Coilia nasus* in Xinjiang River of Jiangxi Province as determined by otolith microchemistry. *Journal of Fishery Sciences of China*, 22, 978—985.
37. Xiao, X., Wang, Y.Y.＆Zhang, H. (2015) Trophic position and its impact on fish in Rao River during the dry season, Jiangxi Province. *Acta Ecologica Sinica*, 35, 6216-6223.
38. Huang, L.L.＆Wu, Z.Q. (2010) Fish resource of Jiulingshan Nature Reserve in Jiangxi Province. *Sichuan Journal of Zoology*, 29, 307-310.
39. Liu, B.B. (2010) Genetic diversity and resources of the four major Chinese carps in the Lower reaches of Xiu River. *Nanchang University*.
40. Zhou, H., Xi, H.B.＆Hu, M.L. (2013) Preliminary survey of fish in Yunjushan Nature Reserve, Jiangxi Province. *Jiangxi Fishery Sciences* *and Technology*, 136, 20-23.
