# Supplementary material for: Biodiversity pattern of fish assemblages in Poyang Lake Basin: Threat and conservation
Source: Ecol Evol. 2019 Sep 26;9(20):11672–83. doi: 10.1002/ece3.5661 (PMC6822132; doi:10.1002/ece3.5661)
Supplement: Supplementary file 4 [file ECE3-9-11672-s004.docx]

**Table S2** Species occurrence in Poyang Lake Basin during the historical period (1980-2000). 1: the species is native to the basin and was present during the considered period, 0: the species is absent from the basin.

|  | Poyang lake | Ganjiang River | Fuhe River | Xinjiang River | Raohe River | Xiuhe River |
| --- | --- | --- | --- | --- | --- | --- |
| *Acipenser sinensis* | 1 | 1 | 0 | 0 | 0 | 0 |
| *Psephurus gladius* | 1 | 0 | 0 | 0 | 0 | 0 |
| *Tenualosa reevesii* | 1 | 1 | 0 | 1 | 0 | 0 |
| *Coilia nasus* | 1 | 1 | 1 | 1 | 0 | 0 |
| *Coilia brachygnathus* | 1 | 1 | 1 | 1 | 0 | 0 |
| *Anguilla japonica* | 1 | 1 | 1 | 1 | 1 | 0 |
| *Zacco platypus* | 1 | 1 | 1 | 1 | 1 | 1 |
| *Opsariichthys bidens* | 1 | 1 | 1 | 1 | 1 | 1 |
| *Aphyocypris chinensis* | 0 | 1 | 0 | 0 | 0 | 0 |
| *Rhynchocypris lagowskii* | 1 | 1 | 0 | 1 | 1 | 0 |
| *Rhynchocypris oxycephalus* | 1 | 1 | 0 | 0 | 0 | 0 |
| *Mylopharyngododon piceus* | 1 | 1 | 1 | 1 | 1 | 1 |
| *Ctenopharyngodon* *idella* | 1 | 1 | 1 | 1 | 1 | 1 |
| *Ochetobius* *elongatus* | 1 | 1 | 1 | 1 | 1 | 1 |
| *Luciobrama* *macrocephalus* | 1 | 1 | 1 | 1 | 0 | 0 |
| *Elopichthys bambusa* | 1 | 1 | 1 | 1 | 1 | 1 |
| *Squaliobarbus* *curriculus* | 1 | 1 | 1 | 1 | 1 | 1 |
| *Hemiculter leucisculus* | 1 | 1 | 1 | 1 | 1 | 1 |
| *Hemiculter bleekeri* | 1 | 1 | 1 | 1 | 1 | 1 |
| *Hemiculter* *tchangi* | 0 | 0 | 1 | 0 | 0 | 0 |
| *Hemiculterella* *sauvagei* | 1 | 1 | 1 | 1 | 0 | 0 |
| *Pseudohemiculter* *dispar* | 1 | 1 | 1 | 1 | 1 | 0 |
| *Pseudohemiculter* *hainanensis* | 0 | 1 | 1 | 1 | 1 | 0 |
| *Pseudolaubuca sinensis* | 1 | 1 | 1 | 1 | 1 | 1 |
| *Pseudolaubuca engraulis* | 1 | 1 | 0 | 0 | 0 | 0 |
| *Toxabramis swinhonis* | 1 | 1 | 1 | 1 | 1 | 0 |
| *Sinibrama* *wui* | 1 | 1 | 0 | 1 | 1 | 0 |
| *Sinibrama* *macrops* | 0 | 1 | 1 | 1 | 1 | 0 |
| *Chanodichthys erythropterus* | 1 | 1 | 1 | 1 | 1 | 1 |
| *Culter alburnus* | 1 | 1 | 1 | 1 | 1 | 1 |
| *Chanodichthys mongolicus* | 1 | 1 | 1 | 1 | 1 | 1 |
| *Chanodichthys dabryi* | 1 | 1 | 1 | 1 | 1 | 1 |
| *Chanodichthys oxycephalus* | 1 | 1 | 0 | 0 | 0 | 0 |
| *Culter oxycephaloides* | 1 | 1 | 1 | 1 | 1 | 0 |
| *Parabramis pekinensis* | 1 | 1 | 1 | 1 | 1 | 1 |
| *Megalobrama mantschuricus* | 1 | 0 | 0 | 0 | 0 | 0 |
| *Megalobrama* *terminalis* | 1 | 1 | 1 | 1 | 1 | 1 |
| *Megalobrama amblycephala* | 1 | 1 | 1 | 1 | 1 | 1 |
| *Xenocypris macrolepis* | 1 | 1 | 1 | 1 | 1 | 1 |
| *Xenocypris davidi* | 1 | 1 | 1 | 1 | 1 | 1 |
| *Plagiognathops microlepis* | 1 | 1 | 1 | 1 | 1 | 1 |
| *Distoechodon* *tumirostris* | 1 | 1 | 1 | 1 | 1 | 1 |
| *Pseudobrama simoni* | 1 | 1 | 1 | 1 | 1 | 1 |
| *Hypophthalmichthys molitrix* | 1 | 1 | 1 | 1 | 1 | 1 |
| *Hypophthalmichthys nobilis* | 1 | 1 | 1 | 1 | 1 | 1 |
| *Abbottina rivularis* | 1 | 1 | 1 | 1 | 1 | 1 |
| *Abbottina* *obtusirostris* | 0 | 1 | 0 | 0 | 0 | 0 |
| *Pseudorasbora parva* | 1 | 1 | 1 | 1 | 1 | 1 |
| *Pseudorasbora elongata* | 1 | 0 | 0 | 0 | 0 | 0 |
| *Pseudogobio* *vaillanti* | 0 | 1 | 1 | 1 | 0 | 1 |
| *Pseudogobio* *guilinensis* | 0 | 1 | 0 | 0 | 0 | 0 |
| *Hemibarbus labeo* | 1 | 1 | 1 | 1 | 1 | 1 |
| *Hemibarbus maculatus* | 1 | 1 | 1 | 1 | 1 | 1 |
| *Hemibarbus* *longirostris* | 0 | 1 | 0 | 1 | 0 | 1 |
| *Hemibarbus* *umbrifer* | 1 | 1 | 0 | 1 | 0 | 0 |
| *Huigobio* *chenhsienensis* | 0 | 1 | 0 | 1 | 0 | 1 |
| *Paracanthobrama guichenoti* | 1 | 1 | 1 | 1 | 0 | 0 |
| *Sarcocheilichthys sinensis* | 1 | 1 | 1 | 1 | 1 | 1 |
| *Sarcocheilichthys parvus* | 1 | 1 | 1 | 1 | 0 | 0 |
| *Sarcocheilichthys kiangsiensis* | 1 | 1 | 1 | 1 | 1 | 1 |
| *Sarcocheilichthys nigripinnis* | 1 | 1 | 1 | 1 | 1 | 1 |
| *Squalidus argentatus* | 1 | 1 | 1 | 1 | 1 | 1 |
| *Squalidus nitens* | 1 | 0 | 0 | 0 | 0 | 0 |
| *Squalidus wolterdstorffi* | 1 | 1 | 1 | 1 | 0 | 0 |
| *Rhinogobio typus* | 1 | 1 | 1 | 1 | 1 | 1 |
| *Rhinogobio cylindricus* | 1 | 1 | 0 | 0 | 0 | 0 |
| *Rhinogobio ventralis* | 0 | 1 | 0 | 0 | 0 | 0 |
| *Platysmacheilus* *exiguus* | 0 | 1 | 1 | 1 | 0 | 0 |
| *Platysmacheilus* *longibarbatus* | 0 | 1 | 0 | 1 | 0 | 0 |
| *Platysmacheilus* *nudiventris* | 0 | 0 | 1 | 0 | 0 | 0 |
| *Gnathopogon imberbis* | 1 | 0 | 0 | 0 | 0 | 0 |
| *Gnathopogon* *tsinanensis* | 0 | 0 | 0 | 1 | 0 | 0 |
| *Gnathopogon taeniellus* | 0 | 0 | 0 | 0 | 1 | 1 |
| *Saurogobio dabryi* | 1 | 1 | 1 | 1 | 1 | 1 |
| *Saurogobio dumerili* | 1 | 1 | 1 | 1 | 0 | 1 |
| *Saurogobio gymnocheilus* | 1 | 1 | 0 | 1 | 0 | 1 |
| *Saurogobio* *gracilicaudatus* | 0 | 1 | 0 | 1 | 0 | 0 |
| *Saurogobio* *xiangjiangensis* | 0 | 1 | 0 | 0 | 0 | 0 |
| *Coreius heterodon* | 1 | 1 | 1 | 1 | 1 | 1 |
| *Coreius septentrionalis* | 1 | 0 | 0 | 0 | 0 | 0 |
| *Microphysogobio* *elongatus* | 0 | 1 | 0 | 0 | 0 | 0 |
| *Microphysogobio* *tungtingensis* | 1 | 1 | 0 | 1 | 0 | 0 |
| *Microphysogobio* *kiatingensis* | 0 | 1 | 1 | 0 | 0 | 0 |
| *Microphysogobio fukiensis* | 0 | 1 | 1 | 1 | 0 | 0 |
| *Gobiobotia filifer* | 1 | 1 | 1 | 0 | 1 | 1 |
| *Gobiobotia* *longibarba* | 0 | 1 | 1 | 1 | 0 | 0 |
| *Gobiobotia* *meridionalis* | 0 | 1 | 1 | 1 | 0 | 0 |
| *Gobiobotia* *tungi* | 0 | 1 | 1 | 1 | 0 | 0 |
| *Acheilognathus macropterus* | 1 | 1 | 1 | 1 | 1 | 1 |
| *Acheilognathus* *barbatus* | 0 | 0 | 1 | 1 | 0 | 0 |
| *Acheilognathus gracilis* | 1 | 1 | 1 | 1 | 0 | 0 |
| *Acheilognathus* *polylepis* | 0 | 1 | 1 | 1 | 0 | 0 |
| *Acheilognathus chankaensis* | 1 | 1 | 1 | 1 | 1 | 1 |
| *Acheilognathus tonkinensis* | 1 | 1 | 1 | 1 | 1 | 1 |
| *Acheilognathus barbatulus* | 1 | 1 | 1 | 1 | 1 | 1 |
| *Acheilognathus hypselonotus* | 1 | 1 | 0 | 0 | 0 | 0 |
| *Acheilognathus tabira* | 1 | 0 | 0 | 1 | 0 | 0 |
| *Acheilognathus elongatus* | 1 | 0 | 1 | 1 | 0 | 1 |
| *Acheilognathus* *peihoensis* | 1 | 1 | 1 | 1 | 0 | 0 |
| *Acheilognathus* *taenianalis* | 1 | 1 | 1 | 1 | 0 | 1 |
| *Acheilognathus imberbis* | 1 | 1 | 0 | 0 | 0 | 1 |
| *Acheilognathus meridianus* | 1 | 1 | 1 | 1 | 0 | 0 |
| *Tanakia himantegus* | 1 | 1 | 0 | 0 | 0 | 0 |
| *Rhodeus ocellatus* | 1 | 1 | 1 | 1 | 1 | 1 |
| *Rhodeus.lighti* | 1 | 1 | 1 | 0 | 0 | 0 |
| *Rhodeus.fangi* | 1 | 0 | 1 | 0 | 0 | 0 |
| *Folifer* *brevifilis* | 1 | 1 | 0 | 1 | 1 | 1 |
| *Acrossocheilus fasciatus* | 1 | 1 | 0 | 0 | 0 | 0 |
| *Acrossocheilus paradoxus* | 1 | 1 | 1 | 0 | 1 | 0 |
| *Acrossocheilus* *hemispinus* | 0 | 1 | 1 | 1 | 1 | 0 |
| *Acrossocheilus* *parallens* | 0 | 1 | 1 | 1 | 1 | 1 |
| *Acrossocheilus* *kreyenbergii* | 0 | 0 | 1 | 1 | 0 | 0 |
| *Onychostoma elongatum* | 0 | 1 | 0 | 1 | 1 | 0 |
| *Spinibarbus* *sinensis* | 1 | 1 | 0 | 1 | 1 | 0 |
| *Spinibarbus hollandi* | 1 | 1 | 1 | 1 | 0 | 0 |
| *Onychostoma* *simum* | 0 | 1 | 0 | 0 | 0 | 0 |
| *Onychostoma* *barbatulum* | 1 | 1 | 1 | 1 | 1 | 1 |
| *Onychostoma* *lini* | 0 | 1 | 0 | 0 | 1 | 0 |
| *Onychostoma rarum* | 1 | 1 | 0 | 0 | 0 | 1 |
| *Barbodes semifasciolatus* | 0 | 1 | 0 | 0 | 0 | 0 |
| *Carassius auratus* | 1 | 1 | 1 | 1 | 1 | 1 |
| *Cyprinus carpio* | 1 | 1 | 1 | 1 | 1 | 1 |
| *Garra* *orientalis* | 1 | 1 | 0 | 0 | 0 | 0 |
| *Parasinilabeo* *assimilis* | 0 | 0 | 0 | 0 | 0 | 1 |
| *Pseudogyrinocheilus* *prochilus* | 0 | 1 | 0 | 0 | 0 | 0 |
| *Myxocyprinus asiaticus* | 1 | 0 | 0 | 1 | 0 | 0 |
| *Cobitis* *taenia* | 1 | 1 | 1 | 1 | 1 | 1 |
| *Cobitis* *sinensis* | 1 | 1 | 0 | 0 | 0 | 0 |
| *Cobitis* *macrostigma* | 1 | 1 | 1 | 1 | 1 | 1 |
| *Misgurnus* *anguillicaudatus* | 1 | 1 | 1 | 1 | 1 | 1 |
| *Paramisgurnus* *dabryanus* | 1 | 1 | 1 | 1 | 0 | 0 |
| *Lepturichthys* *fimbriata* | 1 | 1 | 1 | 1 | 0 | 0 |
| *Schistura* *fasciolata* | 0 | 1 | 0 | 0 | 0 | 0 |
| *Schistura* *incerta* | 0 | 1 | 0 | 0 | 0 | 0 |
| *Leptobotia pellegrini* | 1 | 1 | 0 | 0 | 0 | 0 |
| *Leptobotia* *taeniops* | 1 | 1 | 0 | 1 | 0 | 0 |
| *Leptobotia* *elongata* | 1 | 1 | 0 | 0 | 0 | 0 |
| *Leptobotia* *tchangi* | 0 | 1 | 0 | 0 | 0 | 0 |
| *Leptobotia* *tientainensis* | 0 | 1 | 0 | 0 | 0 | 0 |
| *Parabotia* *banarescui* | 1 | 1 | 0 | 1 | 0 | 0 |
| *Parabotia fasciata* | 1 | 1 | 1 | 1 | 0 | 0 |
| *Parabotia* *kiangsiensis* | 0 | 0 | 0 | 1 | 0 | 0 |
| *Parabotia* *maculosa* | 0 | 1 | 0 | 0 | 1 | 0 |
| *Erromyzon sinensis* | 0 | 0 | 0 | 1 | 0 | 0 |
| *Formosania davidi* | 0 | 0 | 0 | 1 | 0 | 0 |
| Vanmanenia stenosoma | 0 | 1 | 0 | 0 | 0 | 0 |
| *Vanmanenia* *gymnetrus* | 0 | 1 | 0 | 0 | 0 | 0 |
| *Vanmanenia* *pingchowensis* | 0 | 1 | 1 | 0 | 0 | 0 |
| *Pseudogastromyzon* *fasciatus* | 0 | 0 | 0 | 1 | 0 | 0 |
| *Pseudogastromyzon* *changtingensis* | 0 | 1 | 1 | 1 | 0 | 0 |
| *Silurus asotus* | 1 | 1 | 1 | 1 | 1 | 1 |
| *Silurus meridionalis* | 1 | 1 | 0 | 0 | 0 | 0 |
| *Pterocryptis* *cochinchinensis* | 0 | 1 | 0 | 0 | 0 | 0 |
| *Clarias fuscus* | 1 | 1 | 1 | 1 | 1 | 1 |
| *Hemibagrus macropterus* | 1 | 1 | 1 | 1 | 1 | 1 |
| *Tachysurus dumerili* | 1 | 1 | 1 | 0 | 1 | 1 |
| *Pseudobagrus crassilabris* | 1 | 1 | 1 | 1 | 0 | 0 |
| *Pelteobagrus ussuriensis* | 1 | 1 | 1 | 0 | 0 | 0 |
| *Pseudobagrus tenuis* | 1 | 1 | 1 | 0 | 0 | 0 |
| *Pseudobagrus* *ondon* | 0 | 1 | 0 | 0 | 0 | 0 |
| *Pseudobagrus* *analis* | 0 | 1 | 0 | 0 | 0 | 0 |
| *Pseudobagrus pratti* | 1 | 1 | 1 | 1 | 0 | 0 |
| *Pseudobagrus* *taeniatus* | 0 | 1 | 0 | 0 | 0 | 0 |
| *Pseudobagrus* *truncatus* | 0 | 1 | 1 | 1 | 0 | 0 |
| *Tachysurus adiposalis* | 0 | 0 | 0 | 1 | 0 | 0 |
| *Pseudobagrus* *brevicaudatus* | 1 | 1 | 0 | 0 | 0 | 0 |
| *Pseudobagrus albomarginatus* | 1 | 1 | 1 | 1 | 0 | 1 |
| *Tachysurus fulvidraco* | 1 | 1 | 1 | 1 | 1 | 1 |
| *Pseudobagrus vachellii* | 1 | 1 | 1 | 1 | 1 | 1 |
| *Pelteobagrus eupogon* | 1 | 1 | 1 | 0 | 1 | 1 |
| *Tachysurus nitidus* | 1 | 1 | 1 | 0 | 0 | 1 |
| *Liobagrus anguillicauda* | 1 | 1 | 0 | 0 | 0 | 0 |
| *Liobagrus marginatus* | 1 | 1 | 1 | 1 | 0 | 0 |
| *Liobagrus nigricauda* | 1 | 1 | 1 | 0 | 0 | 0 |
| *Liobagrus styani* | 1 | 0 | 0 | 0 | 0 | 0 |
| *Liobagrus* *marginatoides* | 0 | 0 | 1 | 0 | 0 | 0 |
| *Glyptothorax* *fokiensis* | 0 | 1 | 1 | 1 | 0 | 0 |
| *Glyptothorax sinense* | 1 | 1 | 1 | 1 | 0 | 0 |
| *Protosalanx hyalocranius* | 1 | 1 | 1 | 0 | 0 | 0 |
| *Neosalanx oligodontis* | 1 | 0 | 0 | 0 | 0 | 0 |
| *Neosalanx taihuensis* | 1 | 0 | 1 | 1 | 0 | 0 |
| *Hemisalanx brachyrostralis* | 1 | 1 | 1 | 1 | 0 | 0 |
| *Neosalanx* *jordani* | 1 | 0 | 0 | 0 | 0 | 0 |
| *Oryzias latipes* | 1 | 1 | 1 | 1 | 1 | 1 |
| *Hyporhamphus intermedius* | 1 | 1 | 1 | 1 | 0 | 1 |
| *Monopterus albus* | 1 | 1 | 1 | 1 | 1 | 1 |
| *Macrognathus* *aculeatus* | 1 | 1 | 1 | 1 | 1 | 1 |
| *Mastacembelus* *armatus* | 0 | 1 | 0 | 0 | 0 | 0 |
| *Sinobdella sinensis* | 1 | 0 | 0 | 0 | 0 | 0 |
| *Siniperca chuatsi* | 1 | 1 | 1 | 1 | 1 | 1 |
| *Siniperca knerii* | 1 | 1 | 1 | 1 | 1 | 1 |
| *Siniperca* *obscura* | 0 | 1 | 1 | 1 | 1 | 1 |
| *Siniperca roulei* | 1 | 1 | 1 | 1 | 1 | 1 |
| *Siniperca scherzeri* | 1 | 1 | 1 | 1 | 1 | 0 |
| *Siniperca undulata* | 1 | 1 | 0 | 1 | 1 | 0 |
| *Eleotris fusca* | 1 | 0 | 0 | 0 | 0 | 0 |
| *Odontobutis* *sinensis* | 1 | 1 | 1 | 1 | 1 | 1 |
| *Micropercops swinhonis* | 1 | 1 | 1 | 1 | 1 | 1 |
| *Mugilogobius myxodermus* | 1 | 0 | 0 | 1 | 0 | 0 |
| *Rhinogobius cliffordpopei* | 1 | 1 | 0 | 0 | 0 | 0 |
| *Rhinogobius giurinus* | 1 | 1 | 1 | 1 | 0 | 0 |
| *Rhinogobius lindbergi* | 0 | 1 | 0 | 1 | 0 | 0 |
| *Rhinogobius* *brunneus* | 0 | 1 | 0 | 0 | 0 | 0 |
| *Macropodus ocellatus* | 1 | 1 | 1 | 1 | 1 | 1 |
| *Macropodus opercularis* | 1 | 1 | 1 | 1 | 1 | 1 |
| *Channa argus* | 1 | 1 | 1 | 1 | 1 | 1 |
| *Channa asiatica* | 1 | 1 | 1 | 0 | 0 | 0 |
| *Channa* *maculata* | 0 | 1 | 0 | 0 | 0 | 0 |
| *Cynoglossus gracilis* | 1 | 1 | 0 | 1 | 0 | 0 |
| *Cynoglossus abbreviatus* | 1 | 0 | 0 | 0 | 0 | 0 |
| *Takifugu ocellatus* | 1 | 1 | 0 | 0 | 0 | 0 |
| *Takifugu obscurus* | 1 | 1 | 0 | 1 | 0 | 1 |
