# Supplementary material for: Biodiversity pattern of fish assemblages in Poyang Lake Basin: Threat and conservation
Source: Ecol Evol. 2019 Sep 26;9(20):11672–83. doi: 10.1002/ece3.5661 (PMC6822132; doi:10.1002/ece3.5661)
Supplement: Supplementary file 5 [file ECE3-9-11672-s005.docx]

**Table S3** Species occurrence in the Poyang Lake Basin during the current period (2000-2017). 1: the species is native to the basin and was present during the considered period; 0: the species is absent from the basin.

|  | Poyang Lake | Ganjiang River | Fuhe River | Xinjiang River | Raohe River | Xiuhe River |
| --- | --- | --- | --- | --- | --- | --- |
| *Acipenser sinensis* | 1 | 0 | 0 | 0 | 0 | 0 |
| *Psephurus gladius* | 0 | 0 | 0 | 0 | 0 | 0 |
| *Tenualosa reevesii* | 0 | 0 | 0 | 0 | 0 | 0 |
| *Coilia nasus* | 1 | 0 | 0 | 1 | 0 | 0 |
| *Coilia brachygnathus* | 1 | 1 | 0 | 0 | 1 | 1 |
| *Anguilla japonica* | 1 | 1 | 0 | 0 | 0 | 0 |
| *Zacco platypus* | 1 | 1 | 1 | 1 | 1 | 1 |
| *Opsariichthys bidens* | 1 | 1 | 1 | 1 | 1 | 1 |
| *Aphyocypris chinensis* | 0 | 0 | 0 | 0 | 0 | 0 |
| *Rhynchocypris lagowskii* | 0 | 0 | 0 | 0 | 0 | 0 |
| *Rhynchocypris oxycephalus* | 0 | 0 | 0 | 0 | 0 | 1 |
| *Mylopharyngododon piceus* | 1 | 1 | 1 | 1 | 1 | 1 |
| *Ctenopharyngodon idella* | 1 | 1 | 1 | 1 | 1 | 1 |
| *Ochetobius elongatus* | 1 | 1 | 0 | 1 | 0 | 0 |
| *Luciobrama macrocephalus* | 0 | 0 | 0 | 0 | 0 | 0 |
| *Elopichthys bambusa* | 1 | 1 | 0 | 1 | 0 | 1 |
| *Squaliobarbus curriculus* | 1 | 1 | 1 | 1 | 0 | 1 |
| *Tinca tinca* | 0 | 1 | 0 | 0 | 0 | 0 |
| *Hemiculter leucisculus* | 1 | 1 | 1 | 1 | 1 | 1 |
| *Hemiculter bleekeri* | 1 | 1 | 0 | 0 | 0 | 1 |
| *Hemiculter lucidus* | 0 | 0 | 0 | 0 | 0 | 0 |
| *Hemiculter tchangi* | 0 | 0 | 0 | 0 | 0 | 0 |
| *Hemiculterella sauvagei* | 0 | 1 | 0 | 0 | 0 | 0 |
| *Hemiculterella wui* | 0 | 1 | 0 | 0 | 0 | 0 |
| *Pseudohemiculter dispar* | 0 | 1 | 0 | 0 | 1 | 0 |
| *Pseudohemiculter hainanensis* | 0 | 0 | 0 | 0 | 0 | 0 |
| *Pseudolaubuca sinensis* | 1 | 1 | 0 | 1 | 0 | 0 |
| *Pseudolaubuca engraulis* | 1 | 1 | 0 | 0 | 0 | 0 |
| *Toxabramis swinhonis* | 1 | 0 | 0 | 0 | 0 | 0 |
| *Sinibrama wui* | 1 | 1 | 0 | 0 | 0 | 0 |
| *Sinibrama macrops* | 0 | 1 | 1 | 0 | 1 | 1 |
| *Chanodichthys erythropterus* | 1 | 1 | 0 | 0 | 1 | 1 |
| *Ancherythroculter kurematsui* | 0 | 1 | 0 | 0 | 0 | 0 |
| *Culter alburnus* | 1 | 1 | 1 | 1 | 1 | 1 |
| *Chanodichthys mongolicus* | 1 | 1 | 1 | 1 | 1 | 1 |
| *Chanodichthys dabryi* | 1 | 1 | 0 | 0 | 1 | 0 |
| *Chanodichthys oxycephalus* | 1 | 1 | 0 | 0 | 0 | 0 |
| *Culter oxycephaloides* | 0 | 0 | 0 | 0 | 0 | 0 |
| *Parabramis pekinensis* | 1 | 1 | 1 | 1 | 1 | 1 |
| *Megalobrama mantschuricus* | 1 | 0 | 0 | 0 | 1 | 0 |
| *Megalobrama terminalis* | 0 | 1 | 0 | 0 | 0 | 0 |
| *Megalobrama amblycephala* | 1 | 1 | 1 | 1 | 1 | 0 |
| *Xenocypris macrolepis* | 1 | 1 | 1 | 1 | 1 | 1 |
| *Xenocypris davidi* | 1 | 1 | 1 | 1 | 1 | 1 |
| *Plagiognathops microlepis* | 1 | 1 | 1 | 1 | 0 | 0 |
| *Distoechodon tumirostris* | 0 | 1 | 0 | 0 | 0 | 1 |
| *Pseudobrama simoni* | 1 | 1 | 0 | 0 | 1 | 0 |
| *Hypophthalmichthys molitrix* | 1 | 1 | 1 | 1 | 1 | 1 |
| *Hypophthalmichthys nobilis* | 1 | 1 | 1 | 1 | 1 | 1 |
| *Abbottina rivularis* | 1 | 1 | 1 | 0 | 1 | 1 |
| *Abbottina obtusirostris* | 0 | 0 | 0 | 0 | 0 | 0 |
| *Pseudorasbora parva* | 1 | 1 | 1 | 1 | 1 | 1 |
| *Pseudorasbora elongata* | 1 | 0 | 0 | 0 | 0 | 0 |
| *Pseudogobio vaillanti* | 1 | 1 | 1 | 0 | 0 | 1 |
| *Pseudogobio guilinensis* | 0 | 0 | 0 | 0 | 0 | 0 |
| *Belligobio nummifer* | 1 | 1 | 0 | 0 | 0 | 0 |
| *Hemibarbus labeo* | 1 | 1 | 0 | 1 | 1 | 1 |
| *Hemibarbus maculatus* | 1 | 1 | 1 | 1 | 1 | 1 |
| *Hemibarbus longirostris* | 0 | 0 | 0 | 0 | 0 | 0 |
| *Hemibarbus umbrifer* | 0 | 0 | 0 | 0 | 0 | 0 |
| *Huigobio chenhsienensis* | 0 | 0 | 0 | 0 | 0 | 1 |
| *Paracanthobrama guichenoti* | 1 | 0 | 0 | 0 | 0 | 0 |
| *Sarcocheilichthys sinensis* | 1 | 1 | 1 | 0 | 1 | 1 |
| *Sarcocheilichthys parvus* | 1 | 1 | 1 | 0 | 0 | 0 |
| *Sarcocheilichthys kiangsiensis* | 1 | 1 | 1 | 0 | 0 | 1 |
| *Sarcocheilichthys nigripinnis* | 1 | 1 | 1 | 1 | 1 | 1 |
| *Squalidus argentatus* | 1 | 1 | 1 | 0 | 1 | 1 |
| *Squalidus atromaculatus* | 0 | 0 | 0 | 0 | 0 | 1 |
| *Squalidus chankaensis* | 0 | 0 | 0 | 0 | 0 | 0 |
| *Squalidus nitens* | 0 | 0 | 0 | 0 | 0 | 0 |
| *Squalidus wolterdstorffi* | 1 | 0 | 0 | 0 | 0 | 0 |
| *Rhinogobio typus* | 1 | 1 | 1 | 1 | 1 | 1 |
| *Rhinogobio cylindricus* | 1 | 0 | 0 | 0 | 0 | 0 |
| *Rhinogobio ventralis* | 0 | 0 | 0 | 0 | 0 | 0 |
| *Platysmacheilus exiguus* | 0 | 1 | 0 | 0 | 0 | 0 |
| *Platysmacheilus longibarbatus* | 0 | 0 | 0 | 0 | 0 | 0 |
| *Platysmacheilus nudiventris* | 0 | 0 | 0 | 0 | 0 | 0 |
| *Gnathopogon imberbis* | 0 | 1 | 1 | 1 | 0 | 1 |
| *Gnathopogon tsinanensis* | 0 | 0 | 0 | 0 | 0 | 0 |
| *Gnathopogon taeniellus* | 0 | 0 | 0 | 0 | 0 | 0 |
| *Saurogobio dabryi* | 1 | 1 | 1 | 1 | 1 | 1 |
| *Saurogobio dumerili* | 1 | 1 | 0 | 0 | 0 | 0 |
| *Saurogobio gymnocheilus* | 1 | 0 | 0 | 0 | 1 | 0 |
| *Saurogobio gracilicaudatus* | 0 | 0 | 0 | 0 | 0 | 0 |
| *Saurogobio xiangjiangensis* | 0 | 1 | 0 | 0 | 0 | 0 |
| *Coreius heterodon* | 1 | 1 | 0 | 0 | 0 | 0 |
| *Coreius septentrionalis* | 0 | 0 | 0 | 0 | 0 | 0 |
| *Microphysogobio elongatus* | 0 | 0 | 0 | 0 | 0 | 0 |
| *Microphysogobio tungtingensis* | 0 | 0 | 0 | 0 | 0 | 1 |
| *Microphysogobio kiatingensis* | 0 | 0 | 0 | 0 | 0 | 0 |
| *Microphysogobio fukiensis* | 0 | 1 | 0 | 0 | 0 | 0 |
| *Gobiobotia filifer* | 1 | 1 | 0 | 0 | 0 | 0 |
| *Gobiobotia longibarba* | 0 | 0 | 0 | 0 | 0 | 0 |
| *Gobiobotia meridionalis* | 0 | 0 | 0 | 0 | 0 | 0 |
| *Gobiobotia tungi* | 0 | 0 | 0 | 0 | 0 | 0 |
| *Acheilognathus macropterus* | 1 | 1 | 0 | 0 | 1 | 1 |
| *Acheilognathus barbatus* | 0 | 0 | 0 | 0 | 0 | 0 |
| *Acheilognathus gracilis* | 1 | 1 | 0 | 0 | 0 | 0 |
| *Acheilognathus omeiensis* | 0 | 1 | 0 | 0 | 0 | 0 |
| *Acheilognathus polylepis* | 0 | 1 | 0 | 0 | 0 | 0 |
| *Acheilognathus chankaensis* | 1 | 1 | 1 | 0 | 0 | 1 |
| *Acheilognathus tonkinensis* | 1 | 1 | 0 | 0 | 0 | 0 |
| *Acheilognathus barbatulus* | 1 | 1 | 1 | 0 | 0 | 1 |
| *Acheilognathus hypselonotus* | 0 | 1 | 0 | 0 | 0 | 0 |
| *Acheilognathus tabira* | 1 | 0 | 0 | 0 | 0 | 0 |
| *Acheilognathus elongatus* | 0 | 1 | 0 | 0 | 0 | 0 |
| *Acheilognathus peihoensis* | 1 | 1 | 0 | 0 | 0 | 0 |
| *Acheilognathus taenianalis* | 1 | 0 | 0 | 0 | 0 | 1 |
| *Acheilognathus imberbis* | 1 | 1 | 0 | 0 | 0 | 1 |
| *Acheilognathus meridianus* | 0 | 1 | 0 | 0 | 0 | 0 |
| *Tanakia himantegus* | 0 | 1 | 0 | 0 | 0 | 0 |
| *Rhodeus ocellatus* | 1 | 1 | 0 | 1 | 1 | 1 |
| *Rhodeus lighti* | 1 | 1 | 0 | 0 | 0 | 0 |
| *Rhodeus fangi* | 0 | 0 | 0 | 0 | 0 | 0 |
| *Folifer brevifilis* | 0 | 0 | 0 | 0 | 0 | 0 |
| *Acrossocheilus fasciatus* | 0 | 1 | 0 | 0 | 0 | 1 |
| *Acrossocheilus paradoxus* | 0 | 1 | 0 | 0 | 0 | 0 |
| *Acrossocheilus hemispinus* | 0 | 1 | 0 | 0 | 0 | 0 |
| *Acrossocheilus parallens* | 0 | 1 | 1 | 1 | 1 | 1 |
| *Acrossocheilus kreyenbergii* | 0 | 0 | 0 | 0 | 0 | 0 |
| *Onychostoma elongatum* | 0 | 0 | 0 | 0 | 0 | 0 |
| *Spinibarbus denticulatus* | 0 | 1 | 0 | 0 | 0 | 0 |
| *Spinibarbus sinensis* | 0 | 0 | 1 | 1 | 0 | 0 |
| *Spinibarbus hollandi* | 0 | 1 | 0 | 0 | 0 | 1 |
| *Onychostoma simum* | 0 | 0 | 0 | 0 | 0 | 0 |
| *Onychostoma barbatulum* | 0 | 1 | 0 | 1 | 0 | 1 |
| *Onychostoma lini* | 0 | 0 | 0 | 0 | 0 | 0 |
| *Onychostoma rarum* | 0 | 1 | 0 | 0 | 0 | 0 |
| *Barbodes semifasciolatus* | 0 | 0 | 0 | 0 | 0 | 0 |
| *Carassius auratus* | 1 | 1 | 1 | 1 | 1 | 1 |
| *Carassius cuvieri* | 1 | 1 | 0 | 0 | 0 | 1 |
| *Cyprinus carpio* | 1 | 1 | 1 | 1 | 1 | 1 |
| *Garra orientalis* | 0 | 1 | 0 | 0 | 0 | 1 |
| *Parasinilabeo assimilis* | 0 | 0 | 0 | 0 | 0 | 0 |
| *Pseudogyrinocheilus prochilus* | 0 | 0 | 0 | 0 | 0 | 0 |
| *Myxocyprinus asiaticus* | 1 | 1 | 0 | 0 | 0 | 0 |
| *Cobitis taenia* | 0 | 1 | 0 | 0 | 0 | 0 |
| *Cobitis sinensis* | 1 | 1 | 0 | 0 | 1 | 1 |
| *Cobitis macrostigma* | 1 | 1 | 0 | 0 | 0 | 0 |
| *Cobitis sibirica* | 0 | 1 | 0 | 0 | 0 | 0 |
| *Misgurnus anguillicaudatus* | 1 | 1 | 1 | 1 | 1 | 1 |
| *Paramisgurnus dabryanus* | 1 | 1 | 0 | 0 | 0 | 0 |
| *Lepturichthys fimbriata* | 0 | 1 | 0 | 0 | 0 | 0 |
| *Schistura fasciolata* | 0 | 1 | 0 | 0 | 0 | 1 |
| *Schistura incerta* | 0 | 0 | 0 | 0 | 0 | 0 |
| *Sinibotia superciliaris* | 0 | 1 | 1 | 0 | 0 | 0 |
| *Leptobotia pellegrini* | 0 | 0 | 0 | 0 | 0 | 0 |
| *Leptobotia taeniops* | 1 | 1 | 0 | 0 | 0 | 0 |
| *Leptobotia elongata* | 1 | 1 | 0 | 0 | 0 | 0 |
| *Leptobotia tchangi* | 0 | 0 | 0 | 0 | 0 | 0 |
| *Leptobotia tientainensis* | 0 | 0 | 0 | 0 | 0 | 0 |
| *Parabotia banarescui* | 1 | 1 | 0 | 0 | 0 | 0 |
| *Parabotia fasciata* | 1 | 1 | 1 | 1 | 0 | 1 |
| *Parabotia kiangsiensis* | 0 | 1 | 0 | 0 | 0 | 0 |
| *Parabotia maculosa* | 0 | 1 | 0 | 0 | 0 | 0 |
| *Erromyzon sinensis* | 0 | 0 | 0 | 0 | 0 | 0 |
| *Formosania davidi* | 0 | 0 | 0 | 1 | 0 | 0 |
| *Formosania stigmata* | 0 | 1 | 0 | 0 | 0 | 0 |
| *Vanmanenia stenosoma* | 0 | 0 | 0 | 0 | 0 | 0 |
| *Vanmanenia gymnetrus* | 0 | 0 | 0 | 0 | 0 | 0 |
| *Vanmanenia pingchowensis* | 0 | 1 | 0 | 1 | 0 | 1 |
| *Vanmanenia xinyiensis* | 0 | 1 | 0 | 0 | 0 | 0 |
| *Pseudogastromyzon fasciatus* | 0 | 0 | 0 | 0 | 0 | 0 |
| *Pseudogastromyzon changtingensis* | 0 | 1 | 0 | 0 | 0 | 0 |
| *Silurus asotus* | 1 | 1 | 1 | 1 | 1 | 1 |
| *Silurus meridionalis* | 1 | 1 | 0 | 0 | 0 | 0 |
| *Pterocryptis cochinchinensis* | 0 | 0 | 0 | 0 | 0 | 0 |
| *Pterygoplichthys pardalis* | 0 | 1 | 0 | 0 | 0 | 1 |
| *Ictalurus punctatus* | 1 | 1 | 0 | 0 | 0 | 0 |
| *Clarias fuscus* | 1 | 1 | 1 | 1 | 0 | 1 |
| *Clarias gariepinus* | 1 | 1 | 0 | 0 | 1 | 1 |
| *Hemibagrus guttatus* | 0 | 1 | 0 | 0 | 0 | 0 |
| *Hemibagrus macropterus* | 1 | 1 | 1 | 1 | 1 | 1 |
| *Tachysurus dumerili* | 1 | 1 | 0 | 0 | 0 | 0 |
| *Pseudobagrus crassilabris* | 1 | 1 | 0 | 1 | 0 | 0 |
| *Pelteobagrus ussuriensis* | 0 | 0 | 0 | 0 | 0 | 0 |
| *Pseudobagrus tenuis* | 1 | 1 | 1 | 0 | 0 | 1 |
| *Pseudobagrus ondon* | 0 | 1 | 0 | 1 | 0 | 1 |
| *Pseudobagrus analis* | 0 | 1 | 0 | 0 | 0 | 0 |
| *Pseudobagrus pratti* | 0 | 0 | 1 | 0 | 0 | 1 |
| *Pseudobagrus taeniatus* | 0 | 0 | 0 | 0 | 0 | 1 |
| *Pseudobagrus truncatus* | 0 | 0 | 0 | 0 | 0 | 0 |
| *Tachysurus adiposalis* | 0 | 0 | 0 | 0 | 0 | 0 |
| *Pseudobagrus brevicaudatus* | 0 | 0 | 0 | 0 | 0 | 0 |
| *Pseudobagrus albomarginatus* | 1 | 1 | 0 | 0 | 0 | 0 |
| *Tachysurus fulvidraco* | 1 | 1 | 1 | 1 | 1 | 1 |
| *Pseudobagrus vachellii* | 1 | 1 | 0 | 0 | 0 | 0 |
| *Pelteobagrus eupogon* | 1 | 1 | 1 | 0 | 0 | 0 |
| *Tachysurus nitidus* | 1 | 1 | 1 | 0 | 0 | 0 |
| *Liobagrus anguillicauda* | 0 | 1 | 0 | 1 | 0 | 0 |
| *Liobagrus marginatus* | 0 | 0 | 0 | 0 | 0 | 0 |
| *Liobagrus nigricauda* | 1 | 0 | 0 | 0 | 0 | 1 |
| *Liobagrus styani* | 0 | 0 | 0 | 0 | 0 | 0 |
| *Liobagrus marginatoides* | 0 | 0 | 0 | 0 | 0 | 0 |
| *Glyptothorax fokiensis* | 0 | 1 | 0 | 0 | 0 | 0 |
| *Glyptothorax sinense* | 0 | 1 | 0 | 0 | 0 | 1 |
| *Glyptothorax hainanensis* | 0 | 1 | 0 | 0 | 0 | 0 |
| *Protosalanx hyalocranius* | 1 | 0 | 0 | 0 | 0 | 0 |
| *Neosalanx oligodontis* | 0 | 0 | 0 | 0 | 0 | 0 |
| *Neosalanx taihuensis* | 1 | 0 | 0 | 0 | 0 | 0 |
| *Hemisalanx brachyrostralis* | 1 | 0 | 0 | 0 | 0 | 0 |
| *Neosalanx jordani* | 1 | 0 | 0 | 0 | 0 | 0 |
| *Salanx prognathus* | 0 | 1 | 0 | 0 | 0 | 0 |
| *Oryzias latipes* | 1 | 0 | 0 | 0 | 0 | 0 |
| *Hyporhamphus intermedius* | 1 | 1 | 0 | 0 | 0 | 0 |
| *Gambusia affinis* | 0 | 1 | 0 | 0 | 0 | 1 |
| *Monopterus albus* | 1 | 1 | 1 | 1 | 1 | 1 |
| *Macrognathus aculeatus* | 0 | 1 | 0 | 0 | 0 | 0 |
| *Mastacembelus armatus* | 0 | 0 | 0 | 0 | 0 | 0 |
| *Sinobdella sinensis* | 1 | 0 | 0 | 1 | 1 | 1 |
| *Lepomis cyanellus* | 0 | 0 | 0 | 0 | 0 | 1 |
| *Oreochromis niloticus* | 0 | 1 | 0 | 0 | 1 | 1 |
| *Micropterus salmoides* | 1 | 1 | 1 | 1 | 1 | 1 |
| *Siniperca chuatsi* | 1 | 1 | 1 | 1 | 1 | 1 |
| *Siniperca knerii* | 1 | 1 | 1 | 1 | 0 | 1 |
| *Siniperca obscura* | 0 | 1 | 0 | 0 | 0 | 0 |
| *Siniperca roulei* | 1 | 1 | 1 | 0 | 0 | 0 |
| *Siniperca scherzeri* | 1 | 1 | 1 | 1 | 0 | 1 |
| *Siniperca undulata* | 0 | 0 | 0 | 0 | 0 | 1 |
| *Eleotris fusca* | 0 | 0 | 0 | 0 | 0 | 0 |
| *Odontobutis sinensis* | 1 | 1 | 1 | 0 | 1 | 1 |
| *Micropercops swinhonis* | 1 | 1 | 0 | 0 | 0 | 1 |
| *Mugilogobius myxodermus* | 0 | 0 | 0 | 0 | 0 | 0 |
| *Rhinogobius cliffordpopei* | 0 | 1 | 1 | 1 | 0 | 1 |
| *Rhinogobius duospilus* | 0 | 0 | 0 | 0 | 0 | 1 |
| *Rhinogobius giurinus* | 1 | 1 | 1 | 1 | 0 | 1 |
| *Rhinogobius lindbergi* | 0 | 0 | 0 | 0 | 0 | 0 |
| *Rhinogobius leavelli* | 0 | 0 | 0 | 0 | 0 | 0 |
| *Macropodus ocellatus* | 1 | 1 | 1 | 1 | 0 | 1 |
| *Macropodus opercularis* | 1 | 1 | 1 | 0 | 0 | 0 |
| *Channa argus* | 1 | 1 | 1 | 1 | 1 | 1 |
| *Channa asiatica* | 1 | 1 | 1 | 0 | 0 | 1 |
| *Channa maculata* | 0 | 0 | 0 | 0 | 0 | 0 |
| *Cynoglossus gracilis* | 1 | 0 | 0 | 0 | 0 | 0 |
| *Cynoglossus abbreviatus* | 0 | 0 | 0 | 0 | 0 | 0 |
| *Takifugu ocellatus* | 0 | 0 | 0 | 0 | 0 | 0 |
| *Takifugu obscurus* | 0 | 0 | 0 | 0 | 0 | 0 |
