# Supplementary material for: Biodiversity pattern of fish assemblages in Poyang Lake Basin: Threat and conservation
Source: Ecol Evol. 2019 Sep 26;9(20):11672–83. doi: 10.1002/ece3.5661 (PMC6822132; doi:10.1002/ece3.5661)
Supplement: Supplementary file 6 [file ECE3-9-11672-s006.docx]

**Table S4** Ecotype and endangered categories of fish species in Poyang Lake Basin. SE: Settlement fish; MS: Mountain streams fish; M: Migration fish; LL: Lower layer fish; DE: Demersal fish; UL: Upper layer fish; O: Omnivorous; C: Carnivorous; H: Herbivorous; DD: Data Deficient; LC: Least Concern; NT: Near Threatened; VU: Vulnerable; EN: Endangered; CR: Critically Endangered; NE: No evaluation. Division of ecological types of fish was according to Ye and Zhang (2002) and Institute of Hydrobiology, Chinese Academy of Sciences (1976). Life habits were divided into migration, settlement, and mountain streams; feeding habits were divided into herbivorous, carnivorous, and omnivorous; water layer habitats were divided into upper layer, lower layer, and demersal. The Chinese Red List status was according to Jiang et al. (2016).

|  | Ecotype | | | The Chinese Red List status |
| --- | --- | --- | --- | --- |
|  | Feeding habits | Habitat characteristics | Life habits |  |
| *Acipenser sinensis* | O | DE | M | CR |
| *Psephurus gladius* | O | DE | M | CR |
| *Tenualosa reevesii* | O | L | M | CR |
| *Coilia nasus* | C | U | M | LC |
| *Coilia brachygnathus* | C | U | SE | LC |
| *Anguilla japonica* | C | DE | M | EN |
| *Myxocyprinus asiaticus* | O | DE | M | CR |
| *Zacco platypus* | C | U | MS | LC |
| *Opsariichthys bidens* | C | U | MS | LC |
| *Aphyocypris chinensis* | O | L | MS | LC |
| *Rhynchocypris lagowskii* | O | L | MS | LC |
| *Rhynchocypris oxycephalus* | O | L | MS | LC |
| *Mylopharyngododon piceus* | C | DE | M | LC |
| *Ctenopharyngodon* *idella* | H | L | M | LC |
| *Ochetobius* *elongatus* | C | U | M | CR |
| *Luciobrama* *macrocephalus* | C | L | M | CR |
| *Elopichthys bambusa* | C | U | M | LC |
| *Squaliobarbus* *curriculus* | O | L | M | LC |
| *Hemiculter leucisculus* | O | U | SE | LC |
| *Hemiculter bleekeri* | O | U | SE | LC |
| *Hemiculter* *lucidus* | O | U | SE | LC |
| *Hemiculter* *tchangi* | O | U | SE | LC |
| *Hemiculterella* *sauvagei* | O | U | SE | LC |
| *Hemiculterella* *wui* | O | U | SE | LC |
| *Pseudohemiculter* *dispar* | O | U | SE | LC |
| *Pseudohemiculter* *hainanensis* | O | U | SE | LC |
| *Pseudolaubuca sinensis* | O | U | SE | LC |
| *Pseudolaubuca engraulis* | O | U | SE | LC |
| *Toxabramis swinhonis* | O | U | SE | LC |
| *Sinibrama* *wui* | H | L | SE | LC |
| *Sinibrama* *macrops* | H | L | SE | LC |
| *Chanodichthys erythropterus* | C | U | SE | LC |
| *Ancherythroculter* *kurematsui* | C | U | SE | LC |
| *Culter alburnus* | C | U | SE | LC |
| *Chanodichthys mongolicus* | C | U | SE | LC |
| *Chanodichthys dabryi* | C | U | SE | LC |
| *Chanodichthys oxycephalus* | C | U | SE | LC |
| *Culter oxycephaloides* | C | U | SE | LC |
| *Parabramis pekinensis* | H | L | M | LC |
| *Megalobrama mantschuricus* | H | L | SE | LC |
| *Megalobrama* *terminalis* | H | L | SE | LC |
| *Megalobrama amblycephala* | H | L | SE | LC |
| *Xenocypris macrolepis* | H | L | SE | LC |
| *Xenocypris davidi* | H | L | M | LC |
| *Plagiognathops microlepis* | H | L | M | LC |
| *Distoechodon* *tumirostris* | H | L | SE | LC |
| *Pseudobrama simoni* | O | U | M | LC |
| *Hypophthalmichthys molitrix* | H | U | M | LC |
| *Hypophthalmichthys nobilis* | C | U | M | LC |
| *Abbottina rivularis* | O | DE | SE | LC |
| *Abbottina* *obtusirostris* | O | DE | SE | LC |
| *Pseudorasbora parva* | O | L | MS | LC |
| *Pseudorasbora elongata* | O | L | MS | VU |
| *Pseudogobio* *vaillanti* | C | DE | SE | LC |
| *Pseudogobio* *guilinensis* | C | DE | SE | LC |
| *Belligobio* *nummifer* | C | DE | SE | LC |
| *Hemibarbus labeo* | C | DE | SE | LC |
| *Hemibarbus maculatus* | C | DE | SE | LC |
| *Hemibarbus* *longirostris* | C | DE | SE | LC |
| *Hemibarbus* *umbrifer* | C | DE | SE | LC |
| *Huigobio* *chenhsienensis* | O | L | SE | LC |
| *Paracanthobrama guichenoti* | O | L | SE | LC |
| *Sarcocheilichthys sinensis* | O | L | SE | LC |
| *Sarcocheilichthys parvus* | O | L | SE | LC |
| *Sarcocheilichthys kiangsiensis* | O | L | SE | LC |
| *Sarcocheilichthys nigripinnis* | O | L | SE | LC |
| *Squalidus argentatus* | O | L | SE | LC |
| *Squalidus* *atromaculatus* | O | L | SE | DD |
| *Squalidus* *chankaensis* | O | L | SE | DD |
| *Squalidus nitens* | O | L | SE | LC |
| *Squalidus wolterdstorffi* | O | L | SE | LC |
| *Rhinogobio typus* | C | DE | SE | LC |
| *Rhinogobio cylindricus* | C | DE | SE | LC |
| *Rhinogobio ventralis* | C | DE | SE | EN |
| *Platysmacheilus* *exiguus* | C | DE | SE | LC |
| *Platysmacheilus* *longibarbatus* | C | DE | SE | DD |
| *Platysmacheilus* *nudiventris* | C | DE | SE | LC |
| *Gnathopogon imberbis* | O | L | MS | DD |
| *Gnathopogon* *tsinanensis* | O | L | MS | DD |
| *Gnathopogon taeniellus* | O | L | MS | LC |
| *Saurogobio dabryi* | O | DE | SE | LC |
| *Saurogobio dumerili* | O | DE | SE | LC |
| *Saurogobio gymnocheilus* | O | DE | SE | LC |
| *Saurogobio* *gracilicaudatus* | O | DE | SE | LC |
| *Saurogobio* *xiangjiangensis* | O | DE | SE | LC |
| *Coreius heterodon* | O | L | M | LC |
| *Coreius septentrionalis* | O | L | M | CR |
| *Microphysogobio* *elongatus* | C | DE | SE | DD |
| *Microphysogobio* *tungtingensis* | C | DE | SE | DD |
| *Microphysogobio* *kiatingensis* | C | DE | SE | DD |
| *Microphysogobio fukiensis* | C | DE | SE | DD |
| *Gobiobotia filifer* | C | DE | SE | LC |
| *Gobiobotia* *longibarba* | C | DE | SE | DD |
| *Gobiobotia* *meridionalis* | C | DE | SE | DD |
| *Gobiobotia* *tungi* | C | DE | SE | DD |
| *Acheilognathus* *macropterus* | O | L | SE | LC |
| *Acheilognathus* *barbatus* | O | L | SE | LC |
| *Acheilognathus gracilis* | O | L | SE | LC |
| *Acheilognathus* *omeiensis* | O | L | SE | LC |
| *Acheilognathus* *polylepis* | O | L | SE | LC |
| *Acheilognathus* *chankaensis* | O | L | SE | LC |
| *Acheilognathus* *tonkinensis* | O | L | SE | LC |
| *Acheilognathus* *barbatulus* | O | L | SE | LC |
| *Acheilognathus* *hypselonotus* | O | L | SE | LC |
| *Acheilognathus* *tabira* | O | L | SE | LC |
| *Acheilognathus* *elongatus* | O | L | SE | CR |
| *Acheilognathus* *peihoensis* | O | L | SE | DD |
| *Acheilognathus* *taenianalis* | O | L | SE | LC |
| *Acheilognathus imberbis* | O | L | SE | LC |
| *Acheilognathus meridianus* | O | L | SE | LC |
| *Tanakia himantegus* | O | L | SE | NT |
| *Rhodeus ocellatus* | O | L | SE | LC |
| *Rhodeus lighti* | O | L | SE | LC |
| *Rhodeus fangi* | O | L | SE | LC |
| *Folifer* *brevifilis* | O | DE | M | LC |
| *Acrossocheilus fasciatus* | O | L | MS | LC |
| *Acrossocheilus paradoxus* | O | L | MS | LC |
| *Acrossocheilus* *hemispinus* | O | L | MS | LC |
| *Acrossocheilus* *parallens* | O | L | MS | LC |
| *Acrossocheilus* *kreyenbergii* | O | L | MS | LC |
| *Spinibarbus* *denticulatus* | O | L | SE | LC |
| *Spinibarbus* *sinensis* | O | L | SE | LC |
| *Spinibarbus hollandi* | O | L | SE | LC |
| *Onychostoma* *simum* | H | DE | M | LC |
| *Onychostoma* *barbatulum* | H | DE | M | NT |
| *Onychostoma* *lini* | H | DE | M | VU |
| *Onychostoma elongatum* | H | DE | M | DD |
| *Onychostoma rarum* | H | DE | M | VU |
| *Barbodes semifasciolatus* | O | L | MS | LC |
| *Carassius auratus* | O | DE | SE | LC |
| *Cyprinus carpio* | O | DE | SE | LC |
| *Garra* *orientalis* | H | DE | MS | LC |
| *Parasinilabeo* *assimilis* | H | L | SE | VU |
| *Pseudogyrinocheilus* *prochilus* | H | L | MS | LC |
| *Cobitis* *taenia* | O | DE | MS | LC |
| *Cobitis* *sinensis* | O | DE | MS | LC |
| *Cobitis* *macrostigma* | O | DE | MS | LC |
| *Cobitis* *sibirica* | O | DE | MS | LC |
| *Misgurnus* *anguillicaudatus* | O | DE | SE | LC |
| *Paramisgurnus* *dabryanus* | O | DE | SE | LC |
| *Schistura* *fasciolata* | O | DE | MS | DD |
| *Schistura* *incerta* | O | DE | MS | DD |
| *Sinibotia superciliaris* | C | DE | SE | DD |
| *Leptobotia pellegrini* | C | DE | MS | LC |
| *Leptobotia* *taeniops* | O | DE | MS | VU |
| *Leptobotia* *elongata* | C | DE | MS | VU |
| *Leptobotia* *tchangi* | C | DE | MS | DD |
| *Leptobotia* *tientainensis* | C | DE | MS | DD |
| *Parabotia* *banarescui* | C | DE | SE | LC |
| *Parabotia fasciata* | C | DE | SE | LC |
| *Parabotia* *kiangsiensis* | C | DE | SE | DD |
| *Parabotia* *maculosa* | C | DE | SE | LC |
| *Erromyzon sinensis* | O | DE | SE | DD |
| *Lepturichthys* *fimbriata* | H | DE | MS | DD |
| *Formosania davidi* | O | DE | MS | DD |
| *Formosania stigmata* | O | DE | MS | DD |
| *Vanmanenia stenosoma* | O | DE | MS | DD |
| *Vanmanenia* *gymnetrus* | O | DE | MS | DD |
| *Vanmanenia* *pingchowensis* | O | DE | MS | LC |
| *Vanmanenia* *xinyiensis* | O | DE | MS | DD |
| *Pseudogastromyzon* *fasciatus* | O | DE | MS | DD |
| *Pseudogastromyzon* *changtingensis* | O | DE | MS | DD |
| *Silurus asotus* | C | L | SE | LC |
| *Silurus meridionalis* | C | L | SE | LC |
| *Pterocryptis* *cochinchinensis* | C | L | SE | LC |
| *Clarias fuscus* | C | L | MS | LC |
| *Hemibagrus* *guttatus* | O | L | SE | LC |
| *Hemibagrus macropterus* | O | DE | MS | LC |
| *Tachysurus dumerili* | C | L | SE | LC |
| *Pseudobagrus crassilabris* | C | DE | SE | LC |
| *Pelteobagrus ussuriensis* | C | L | SE | LC |
| *Pseudobagrus tenuis* | C | DE | SE | DD |
| *Pseudobagrus* *ondon* | C | DE | SE | DD |
| *Pseudobagrus* *analis* | C | DE | SE | DD |
| *Pseudobagrus pratti* | C | DE | SE | VU |
| *Pseudobagrus* *taeniatus* | C | DE | SE | DD |
| *Pseudobagrus* *truncatus* | C | DE | SE | DD |
| *Tachysurus adiposalis* | C | DE | SE | NT |
| *Pseudobagrus* *brevicaudatus* | C | DE | SE | DD |
| *Pseudobagrus albomarginatus* | C | DE | SE | LC |
| *Tachysurus fulvidraco* | C | DE | SE | LC |
| *Pseudobagrus vachellii* | C | DE | SE | LC |
| *Pelteobagrus eupogon* | C | DE | SE | DD |
| *Tachysurus nitidus* | C | DE | SE | LC |
| *Liobagrus anguillicauda* | O | DE | SE | DD |
| *Liobagrus marginatus* | O | DE | SE | VU |
| *Liobagrus nigricauda* | O | DE | SE | DD |
| *Liobagrus styani* | O | DE | SE | CR |
| *Liobagrus* *marginatoides* | O | DE | SE | DD |
| *Glyptothorax* *fokiensis* | C | DE | SE | LC |
| *Glyptothorax sinense* | C | DE | SE | LC |
| *Glyptothorax hainanensis* | C | DE | SE | DD |
| *Protosalanx hyalocranius* | C | L | SE | DD |
| *Neosalanx oligodontis* | C | L | SE | DD |
| *Neosalanx taihuensis* | C | L | SE | LC |
| *Hemisalanx brachyrostralis* | C | L | SE | VU |
| *Neosalanx* *jordani* | C | L | SE | DD |
| *Salanx prognathus* | C | L | SE | EN |
| *Oryzias latipes* | C | U | MS | LC |
| *Hyporhamphus intermedius* | C | U | SE | LC |
| *Monopterus albus* | C | DE | SE | LC |
| *Macrognathus* *aculeatus* | C | DE | SE | LC |
| *Mastacembelus* *armatus* | C | DE | SE | LC |
| *Sinobdella sinensis* | C | DE | SE | DD |
| *Siniperca chuatsi* | C | U | SE | LC |
| *Siniperca knerii* | C | U | SE | LC |
| *Siniperca* *obscura* | C | U | SE | NT |
| *Siniperca roulei* | C | U | SE | VU |
| *Siniperca scherzeri* | C | U | SE | LC |
| *Siniperca undulata* | C | U | SE | NT |
| *Eleotris fusca* | C | DE | SE | LC |
| *Odontobutis* *sinensis* | C | DE | SE | LC |
| *Micropercops swinhonis* | C | DE | SE | LC |
| *Mugilogobius myxodermus* | C | DE | MS | DD |
| *Rhinogobius* *cliffordpopei* | C | DE | MS | LC |
| *Rhinogobius* *duospilus* | C | DE | MS | DD |
| *Rhinogobius giurinus* | C | DE | MS | LC |
| *Rhinogobius lindbergi* | C | DE | MS | DD |
| *Rhinogobius* *leavelli* | C | DE | MS | LC |
| *Macropodus ocellatus* | C | L | SE | LC |
| *Macropodus opercularis* | C | L | SE | LC |
| *Channa argus* | C | DE | SE | LC |
| *Channa asiatica* | C | DE | SE | LC |
| *Channa* *maculata* | C | DE | SE | LC |
| *Cynoglossus gracilis* | O | DE | M | LC |
| *Cynoglossus abbreviatus* | O | DE | M | LC |
| *Takifugu ocellatus* | C | DE | M | LC |
| *Takifugu obscurus* | C | DE | M | LC |
